# Exploration of acetanilide derivatives of 1-(ω-phenoxyalkyl)uracils as novel inhibitors of Hepatitis C Virus replication

**DOI:** 10.1038/srep29487

**Published:** 2016-07-12

**Authors:** Andrea Magri, Alexander A. Ozerov, Vera L. Tunitskaya, Vladimir T. Valuev-Elliston, Ahmed Wahid, Mario Pirisi, Peter Simmonds, Alexander V. Ivanov, Mikhail S. Novikov, Arvind H. Patel

**Affiliations:** 1 MRC-University of Glasgow Centre for Virus Research, Glasgow, UK; 2 Nuffield Department of Medicine, University of Oxford, Oxford, United Kingdom; 3 Department of Translational Medicine, Università del Piemonte Orientale, Novara, Italy; 4 Department of Pharmaceutical & Toxicological Chemistry, Volgograd State Medical University, Volgograd, Russia; 5 Engelhardt Institute of Molecular Biology, Russian Academy of Science, Moscow, Russia; 6 Department of Biochemistry, Faculty of Pharmacy, Minia, University, Minia, Egypt

## Abstract

Hepatitis C Virus (HCV) is a major public health problem worldwide. While highly efficacious directly-acting antiviral agents have been developed in recent years, their high costs and relative inaccessibility make their use limited. Here, we describe new 1-(ω-phenoxyalkyl)uracils bearing acetanilide fragment in 3 position of pyrimidine ring as potential antiviral drugs against HCV. Using a combination of various biochemical assays and *in vitro* virus infection and replication models, we show that our compounds are able to significantly reduce viral genomic replication, independently of virus genotype, with their IC_50_ values in the nanomolar range. We also demonstrate that our compounds can block *de novo* RNA synthesis and that effect is dependent on a chemical structure of the compounds. A detailed structure-activity relationship revealed that the most active compounds were the N^3^-substituted uracil derivatives containing 6-(4-bromophenoxy)hexyl or 8-(4-bromophenoxy)octyl fragment at N^1^ position.

Hepatitis C Virus (HCV) has infected 150 million people worldwide^1^. It has the propensity to cause chronic infection in the majority of individuals that can lead to liver cirrhosis and hepatocellular carcinoma. It is estimated that HCV causes from 350,000 to 500,000 deaths each year, representing a significant health problem.

HCV is a positive single-strand RNA virus, belonging to the *Hepacivirus* genus in the family *Hepaciviridae*. Its genome is ~9.6 Kb long encoding a single polyprotein of 3000 amino acids that is processed into structural (Core, E1 and E2) and non-structural (p7, NS2, NS3, NS4A, NS4B, NS5A and NS5B) proteins by viral and cellular proteases^2^. The virus particle consists of the RNA genome packaged in a capsid (Core) that is surrounded by a lipid envelope presenting viral glycoproteins (E1 and E2)^3^. Several lipoproteins including apoE, apoC1 and apoB are associated with the virus particle forming a lipid cloak. During infection, the virus initially attaches to the cell through weak interactions with GAGs or LDLR on the cell surface followed by interactions with numerous (co-)receptors; key proteins include SR-B1, CD81, Claudin-1 (CLN1) and Occludin (OCLN)^4^. The virus is then internalized by clathrin-mediated endocytosis and undergoes uncoating following acidification in the early endosomes. The viral genome released into the cytosol is translated by cellular ribosomes into a single polyprotein that is then processed into mature proteins as described above. The incoming RNA is firstly replicated by virus and host proteins into negative-strand RNA that is then used as template to synthesize the progeny of positive-strand RNA. Viral RNA replication occurs in double-membrane vesicles (DMVs) associated with endoplasmic reticulum (ER) where a portion of the freshly synthesized RNA is packaged into nascent particles that acquire their envelope in the ER and are then released from cells likely using the secretory pathway^5^,^6^.

There is no vaccine available to date. Until recently, standard hepatitis C treatments consisted of a combination of PEGylated interferon alpha and Ribavirin for a period of 24 or 48 weeks, depending on HCV genotype (gt). Cure rates were between 70 and 80% for gt 2 and 3, respectively, and 45 to 70% for other genotypes. The first directly acting antiviral agents (DDA) (i.e. inhibitors of HCV NS3/4A protease Telaprevir and Boceprevir) were introduced in 2011. Combining either Boceprevir or Telaprevir with Ribavirin and PEGylated interferon alpha improves antiviral response. Unfortunately, this benefit is limited to hepatitis C gt 1^7^ and is offset by a greater rate of adverse effects, especially in case of patients with liver fibrosis^8^, requiring additional medical surveillance^9^,^10^,^11^.

FDA approved Sofosbuvir in 2013 as a highly active inhibitor of HCV NS5B RNA-dependent RNA polymerase. It is the first drug to be used in combination with Ribavirin for treatment of hepatitis C genotypes 2 and 3 without PEGylated interferon. Sofosbuvir, Simeprevir (also approved by FDA in 2013 as inhibitor of HCV protease) and Ribavirin combination has success rates of around 90% for all viral genotypes^12^. Combinations that contain HCV NS5A inhibitors Daclatasvir and Ledipasvir show even higher success rates of 93 to 100% depending on viral genotype^13^. In the end of 2014 a new cocktail was approved called Viekira Pak that includes Ombitasvir, Paritaprevir, Ritonavir and Dasabuvir^14^. Viekira Pak showed very promising results in the treatment of chronic hepatitis C^15^. However, access to directly acting anti-HCV therapies is severely limited since these regimes are very expensive^16^,^17^; moreover, some drugs have not entered all the regional markets, such as Sofosbuvir in Russia, where the treatment of adults is not covered even by existing drugs. So newly developed drugs may have niche regional markets. In addition, their clinical usage faces development of drug resistant HCV strains^18^,^19^. Hence, the development of novel anti-HCV agents to increase effectiveness, shorten treatment periods and widen availability (and affordability) is an urgent task for the modern healthcare.

Recently, we synthesized a series of 1-[ω-(phenoxy)alkyl]uracil derivatives, which showed potent anti-human cytomegalovirus (HCMV) activity in cell culture^20^. In this paper, we describe the anti-HCV properties of their analogues bearing an amide fragment at position 3 of the uracil residue.

## Results

**Figure 1:**
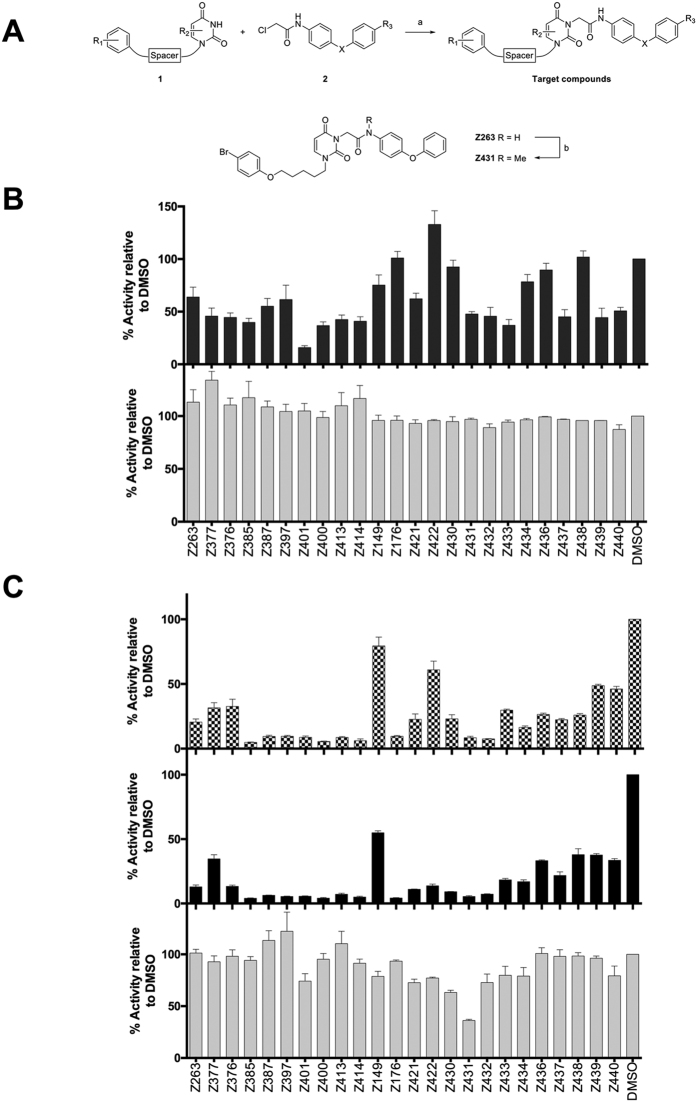
(**A**) Synthesis of the target compounds listed in Table 1. Reagents and conditions: a) K_2_CO_3_, DMF, room temperature, 24 h; b) MeI, NaH, DMF, 0 °C, 4 h. (**B,C**) Antiviral activity on HCVcc and cell viability. (**B**) Huh7-J20 were pretreated for 1 h and then infected with JFH-1 in the presence of the drugs at the concentration of 10 μM for 3 h. Then, inoculum was removed and cells re-fed with fresh medium. After 72 h the infected cell medium was collected and inhibitory effect was determined by measuring SEAP activity (top). Cell viability analysis was performed following same conditions with no viral infection (bottom). The values of DMSO-treated cells are expressed as 100% and those of the drug-treated cells as relative to this DMSO control. Error bars indicate standard error of the means for 3 experiments. (**C**) Huh7-J20 cells were infected for 3 h with JFH-1 and then treated for 3 d with drugs (10 μM) (post-entry, top) or infected for 3 h in the presence of the drugs, after a pretreatment of 1 h, and then incubated for 3 d maintaining drugs in the medium (full life cycle, centre). SEAP assay was performed on infected cell medium to determine antiviral activity. Data are presented as in (**B**) above. Cell viability was performed following full life cycle protocol (bottom).

Compound library was synthesized by alkylation of 1-substituted uracil derivatives **1** with 2-chloroacetanilides **2** as shown in Fig. 1A. Synthesis of the starting uracil derivatives **1** was described previously^20^. 2-Chloro-*N*-(4-phenoxyphenyl)acetamides and their analogues **2** were prepared according to earlier published method^21^. Methylation of Z263 to obtain Z431 was performed as described^22^.

To assess the effect of compounds on HCV infection, we used three protocols: Protocol 1 where Huh7-J20^23^ cells were pre-treated for 1 h and exposed to the cell culture infectious HCV (HCVcc) gt 2a strain JFH-1 in the presence of 10 μM drug or equivalent DMSO (as a vehicle control) for 3 h. The cells were then washed and re-fed with fresh medium (without drug) for 72 h. Thus, this model allows investigating a possible effect on HCV entry. In the second protocol, Huh7-J20 cells were pre-treated, infected with JFH-1 HCVcc for 3 h in the presence of drugs or DMSO and then exposed to the compounds for a further 72 h; this model is commonly used to test effect of compounds on full viral life cycle. In the third protocol, we first infected Huh7-J20 cells with JFH-1 HCVcc for 3 h and replaced the inoculum with fresh medium containing the drug or DMSO to determine if the effect of the drug is exerted post-viral entry (e.g. RNA replication and/or virus assembly). Cell viability assays were performed in parallel. Several compounds showed a good inhibitory effect on virus entry, particularly the compound Z401 which inhibited virus infection by 80% relative to the DMSO control (Fig. 1B). Interestingly, Z401 was also strongly effective post-entry (Fig. 1C, top panel). Most compounds, however, showed a strong antiviral activity on both the post-entry or full life cycle model, with no or little adverse effect on cell viability except compound Z431 (Fig. 1C). Together, these data suggest that most of the compounds affect virus genome replication.

**Figure 2:**
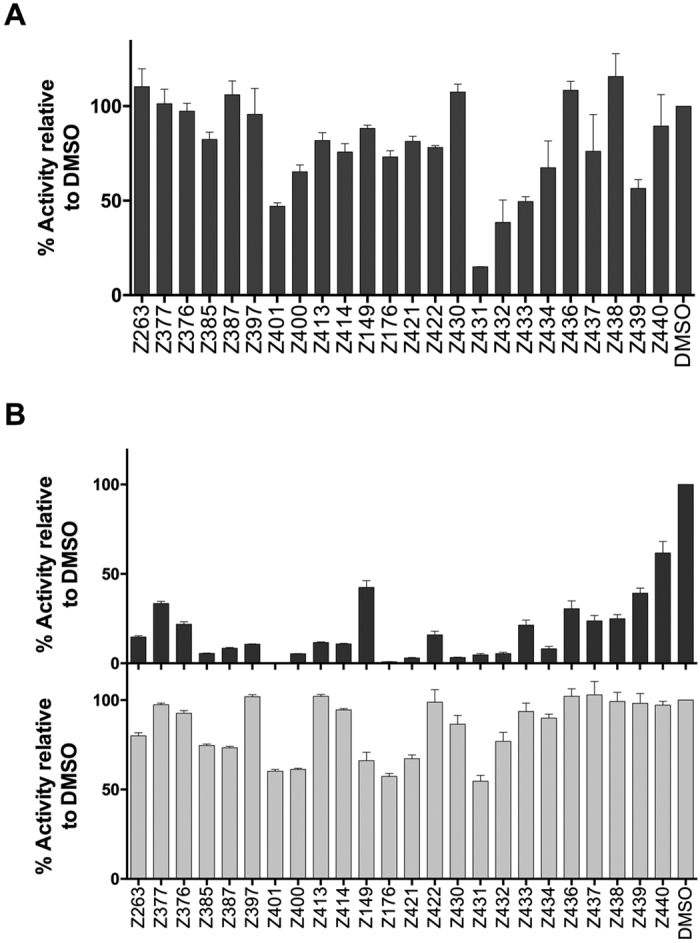
Effect of compounds on virus life cycle. (**A**) Inhibition of HCV pseudoparticle entry. Huh7 were pretreated for 1 h and then infected with HCVpp in the presence of the drugs. After 3 h cells were washed twice and incubated for 72 hours with fresh medium. After 3 d cells were harvested for luciferase assay. The luciferase activity is presented as % relative to that of DMSO-treated cells. (**B**) Inhibitory effect on viral replication. Huh7 cells were electroporated with JFH-luc RNA, seeded in the presence of the drugs at the concentration of 10 μM and then incubated for 24 h. Then, cells were lysed to measure luciferase activity (top). In parallel cell viability was measured (bottom). The number obtained from DMSO-treated cells is expressed as 100%.

To further test the effect on HCV entry, we used the surrogate retrovirus-based pseudoparticle (HCVpp) model. HCVpp consist of retroviral particles, expressing HCV JFH-1 glycoproteins E1 and E2 on their surface and with firefly luciferase as transgene. The results showed that just 2 drugs were able to inhibit HCVpp by more than 50%; Z431 strongly blocked HCV entry (84% inhibition), while Z432 showed a moderate effect (62%) (Fig. 2A). While Z401 was a potent inhibitor of HCVcc entry (80% inhibition) (Fig. 1B), it had a moderate effect on HCVpp infection (50%) (Fig. 2A).

Our results above indicate that most of our compounds target virus replication. To confirm this, all the drugs were tested for their ability to inhibit replication of a viral sub-genomic replicon. Huh7 cells, electroporated with JFH1-luc replicon RNA, were seeded and incubated for 24 h in the presence of the drugs before measuring luciferase signal. Interestingly, almost all the compounds showed a good inhibition of viral RNA replication, with some compounds being able to block up to 95% of replication (Fig. 2B). These data confirm the hypothesis that these compounds are able to inhibit HCV replication as previously observed in HCVcc experiments (Fig. 1C). In this model, the cell viabilities were up to 10% less compared to those in previous experiments, possibly due to an increase in drug uptake resulting from electroporation.

**Figure 3:**
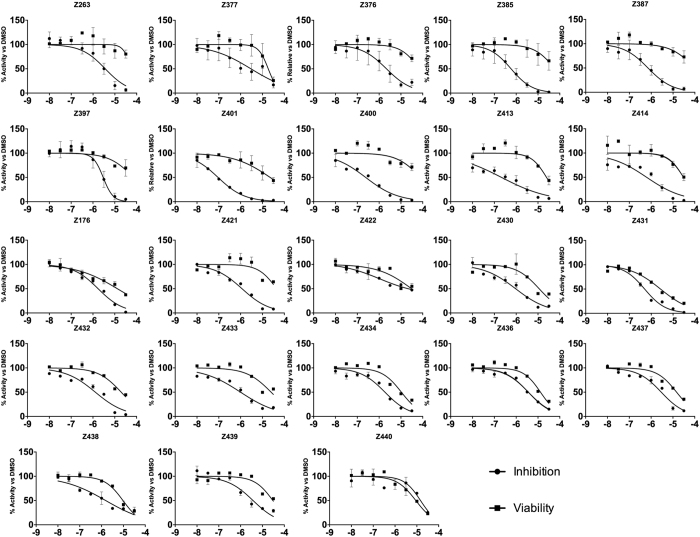
Antiviral activity on a replicon cell line. Huh-J17 were plated in the presence of the drugs (10 μM) and then incubated for 24, 48 or 72 h. Then, cells were harvested and inhibitory effect was measured comparing luciferase levels. DMSO-treated cells value is represented as 100%. Cell viability was measured after 72 hours.

The inhibitory effect above, observed on Huh7 cells transiently transfected with the HCV sub-genomic replicon, was then confirmed using the stable replicon cell line Huh7-J17^24^. Cells were plated in the presence of the drugs and incubated for 24, 48 or 72 h. Results confirmed the previous observation, with a good number of drugs showing high inhibition of the viral replicon RNA in a time-dependent fashion (Fig. 3). Moreover, 9 drugs (Z385, Z387, Z401, Z400, Z176, Z421, Z430, Z431, Z432) showed a strong inhibition, up to 95% compared to DMSO-treated cells. Interestingly, all the compounds induced toxicity effects similar to those observed on electroporated cells.

We next obtained dose-response curves for each compound to determine their IC50 and CC50 values. These experiments were conducted testing all the compounds with concentrations starting at 30 μM and decreasing with 3-fold dilutions. Huh7-J20 cells were treated and infected with HCV following the second protocol described above. IC50 and CC50 values were collected 3 d post-infection (Table 1 and Supplementary Fig. S2). Interestingly, most compounds showed good IC50 values (<5 μM), with some drugs conferring inhibition at nM scale (underlined in the Table 1). Their CC50 values ranged between 2 μM to 60 μM. Considering a SI cut-off of 3, almost all the compounds passed this limit, with many of them showing extremely higher values. Notably, compounds Z385 and Z387, and Z400 and Z401 exhibited SI values >100 and >200, respectively (Table 1). Therefore, these 4 compounds were selected for further evaluation.

To test the antiviral efficacy of these compounds on other HCV genotypes, we performed dose response experiments as described above on cells electroporated with sub-genomic replicons derived from the gt1b Con1^25^ and the gt3a S52^26^,^27^ viral isolates. As shown in Table 2 and Supplementary Fig. S3, all 4 compounds showed strong inhibition on the gt1b replicon, with IC50 values ranging between 2 and 23 μM. Interestingly, a more potent antiviral effect was detected on gt3a replicon, with IC_50_ values ranging between 1 and 0.6 nM (Table 2 and Supplementary Fig. S3). Together, these data strongly indicate that the anti-HCV activity of compounds Z385, Z387, Z400 and Z401 is likely to be pan-genotypic.

**Figure 4:**
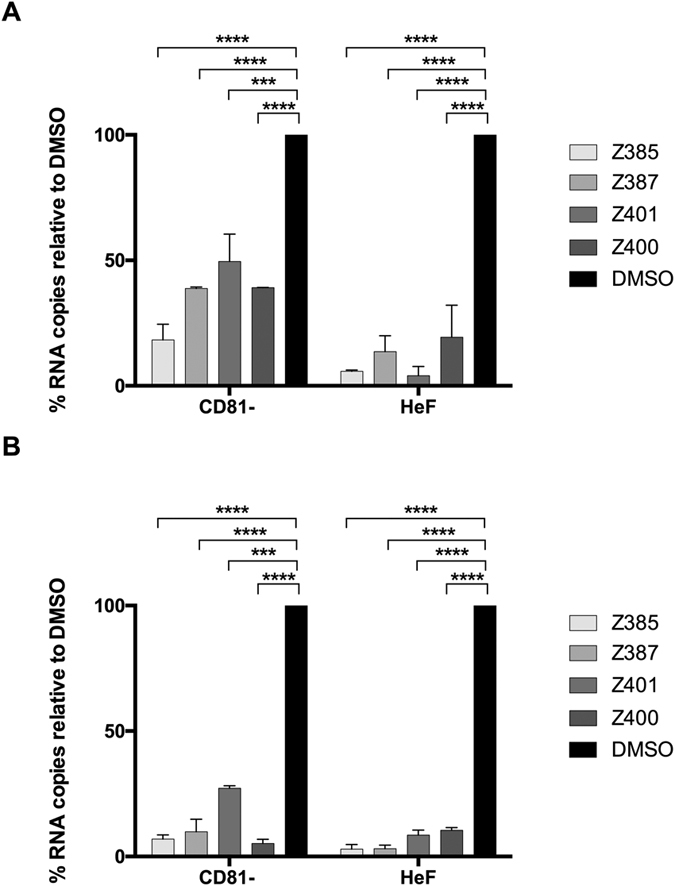
Viral RNA levels in Huh7-Lunet-CD81 and Huh7L-H/EF. Both cell lines were electroporated with JFH-1 RNA, seeded in the presence of the drugs (10 μM) and incubated for 3 d. (**A**) Viral total RNA levels were measured by RT-qPCR. The data are presented as percentage relative to DMSO-treated cells which is expressed as 100%. One-way ANOVA Test: ***p < 0.001; ****p < 0.0001 for both cell lines. (**B**) Viral particle RNA quantification. Infected cell supernatants were collected 72 h post electroporation and digested with RNAse A for 2 h. RNA quantification was performed and data presented as percentage relative to DMSO-treated cell control as described above. One-way ANOVA Test: ***p < 0.001; ****p < 0.0001 for both cell lines.

To rule out the possible effect of secondary infection in our experiments described in Fig. 1 and Table 1, we tested the viral RNA replication inhibitory activity of these compounds in the Huh7-Lunet CD81 cells. These cells do not express CD81, a host factor essential for virus entry^28^. While these cells remain competent for viral RNA replication and assembly upon direct introduction of its genome into the cells, they are refractory to virus entry and spread of infection. As such, these cells can be used to investigate the inhibitory effect of antiviral compounds in a single-cycle infection assay. As control, we used Huh7L-H/EF, a Huh7 Lunet CD81-derived cell line over-expressing human CD81^28^. Both cell lines were electroporated with the gt2a HCVcc JFH-1 RNA, seeded in the presence of the drugs and incubated for 72 h before measuring intracellular RNA levels, and those associated with the viral progeny secreted into the medium of electroporated cells. The latter was performed following digestion with RNAse A to remove any residual untransfected viral RNA. All 4 compounds strongly inhibited virus replication in both cell lines (Fig. 4A). In parallel, we also measured the progeny virus release by quantifying viral RNA into the medium (Fig. 4B). In keeping with the results obtained in Fig. 4A, there was a drastic reduction in virus progeny levels in the medium of cells infected in the presence of all compounds (Fig. 4B). Collectively, these results indicate that compounds Z385, Z387, Z400 and Z401 affect virus RNA replication.

To assess whether our compounds could trigger viral escape, they were evaluated over a long period on the replicon cell line using a concentration of 3 μM, in order to reduce the cytotoxic effect. Huh7-J17 cells were treated with Z385, Z387, Z400, Z401 and DMSO and cultured for 20 days. Replication levels were measured at different time point on the same number of cells. Noteworthy, all the compounds showed a significant level of inhibition, with Z401 able to block up to 90% (Supplementary Fig. S1). Moreover, the antiviral activity was evident through the entire period of the experiment, with no rebound observed.

**Figure 5:**
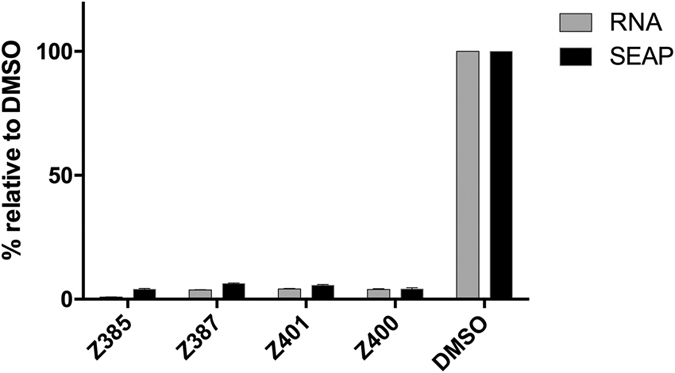
Antiviral activity on cell-culture adaptive mutant JFH-1_DSGCSL_. Huh7 cells were infected following the third protocol with adaptive mutant virus and incubated for 3 d with drugs at the concentration of 10 μM. Viral RNA levels and relative SEAP activity were measured as previously described.

To further confirm antiviral activity, we tested Z385, Z387, Z400, and Z401 on a cell culture adaptive derivative HCV JFH-1_DSGCSL_ strain that consistently replicates to high titres^29^. Huh7 cells were infected with HCVcc JFH-1_DSGCSL_ in the presence of drugs as per Protocol 3 described above and RNA was collected 72 h post-infection. Total RNA and the viral RNA were quantified as above. As shown in Fig. 5, compounds Z385, Z387, Z400 and Z401 reduced the total viral RNA levels by 90%. Similar results were obtained with the parent HCVcc JFH1 strain where a concurrent decrease in virus replication levels was seen in our reporter cell line (Fig. 5).

To explore the possibility that these compounds may affect HCV-encoded enzymes, Z385, Z400 and Z401 were tested for inhibition of recombinant viral NS3 helicase/NTPase and NS5B RNA-dependent RNA polymerase (RdRp) activities. Notably, we tested both primer-dependent and *de novo* polymerase activities of RdRp that was derived from both 1b (isolate Con1) and 2a (isolate JFH1) genotypes. However, none of the compounds tested exhibited any notable inhibitory activity towards either of the viral enzymes (Supplement, Fig. S4). These data suggest that the compounds likely target one or more cellular factors that are critical for virus RNA replication.

**Figure 6:**
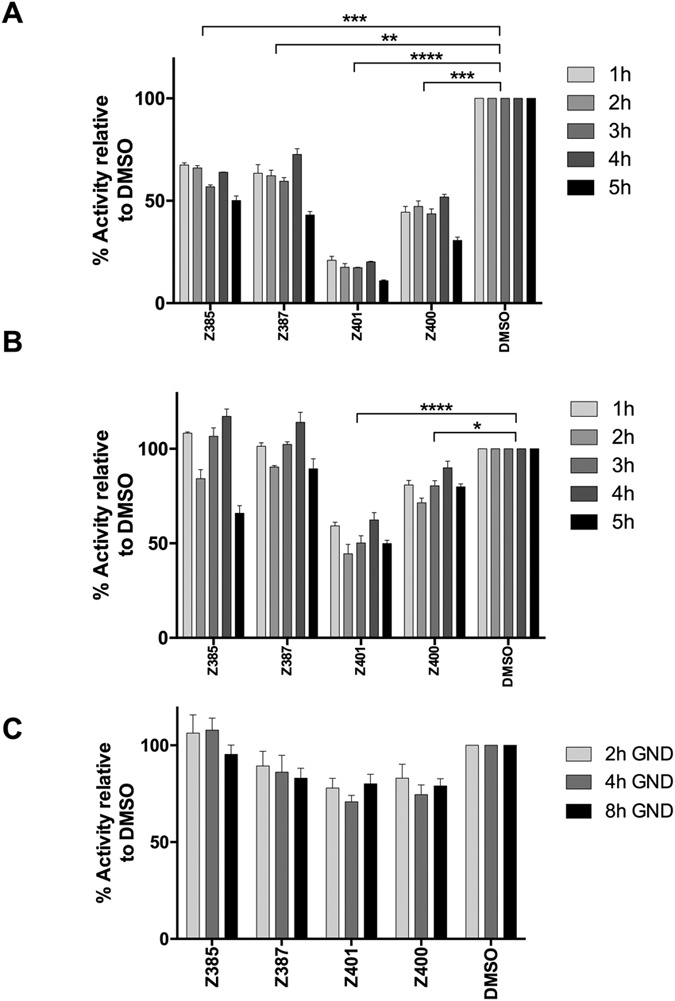
Inhibition at early stage of viral replication. (**A**) Huh7 cells were electroporated with SGR-JFH-luc RNA and seeded in the presence of the drugs for 1 to 5 h. Then, drugs were removed, replaced with fresh medium and incubated for 24. One-way ANOVA Test (**p < 0.01; ***p < 0.001; ****p < 0.0001). (**B**) Huh7 cells were treated as described for A but incubated for 48 h. One-way ANOVA Test (*p < 0.05; ****p < 0.0001). (**C**) Possible effect on viral translation. Cells were electroporated with SGR-JFH-GND-luc and seeded in the presence of drugs. Luciferase was analysed 2, 4 and 8 h post transfection.

**Figure 7:**
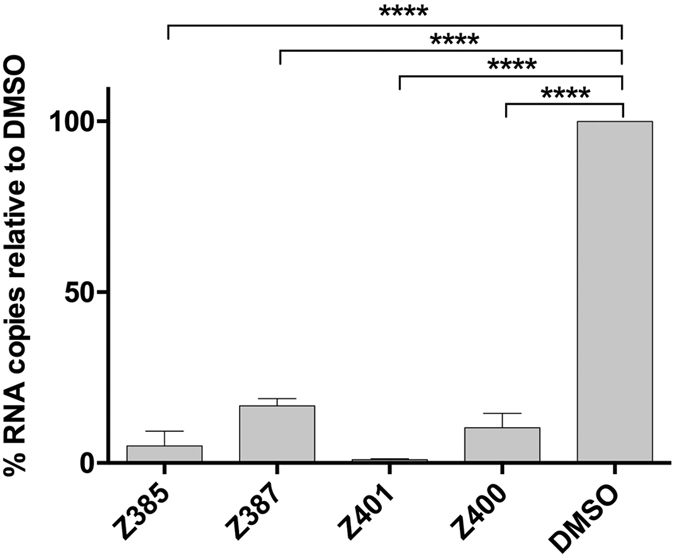
Huh7 cells were infected with TCP expressing N17 SGR-luc and exposed to the compounds for 24 h. Results were obtained measuring luciferase expression from cell lysates. One-way ANOVA Test (****p < 0.0001).

Our data in Figs 1B and 4 suggest that these compounds may be acting at an early stage of viral replication. Compound Z401 in particular showed a partial effect that could be explained by a rapid inhibition of viral replication. To explore this idea, we tested the ability of the 4 selected compounds to inhibit viral replication under a slightly modified condition in which cells were treated only during the first hours after RNA transfection. Briefly, immediately after electroporation with SGR-JFH-luc RNA Huh7 cells were treated with drugs for a time ranging from 1 to 5 h. The cells were then washed, and incubated in fresh medium in the absence of drugs 24 or 48 h. The results at 24 h post transfection, shown in Fig. 6A, confirmed a block of approximately 50% to 60% for Z385, Z387 and Z400 and 80% for Z401. However, data obtained 48 h post transfection showed a massive rebound of viral replication, with no effects observed for all the compounds except a small inhibition in 5 h treatment; only Z401, exhibited a persistent antiviral activity of 50% (Fig. 6B). In parallel, these compounds were tested on Huh7 cells electroporated with the viral NS5B-defective SGR-JFH-GND-luc RNA, a sub-genomic replicon with a mutation in the GDD domain that blocks the viral NS5B polymerase activity and hence RNA replication. However, this mutant RNA is expected to be translation-competent, at least up to 8 h post-transfection. Huh7 cells, electroporated with JFH-GND-luc RNA and plated in the presence of the compounds, showed no significant effects on the levels of luciferase, indicating that viral RNA translation was not affected (Fig. 6C). Moreover, no inhibition was observed when the compounds were tested similarly on the stable replicon cell line (data not shown) which persistently replicates viral RNA. Together, these results indicate that the compounds tested exert their antiviral effect mainly during the initial stage of viral replication. Finally, we tested these compounds using trans-complemented pseudo-typed HCV replicon particles (TCP). Based on a system described previously^30^, we generated TCP encapsidating our N17 subgenomic replicon^24^ in the medium of Huh7-J17 replicon cells that had been transfected with a plasmid construct expressing the VSV-G protein. The TCPs produced thus are capable of infecting and delivering replication-competent N17 replicon into naïve Huh7 cells without generating progeny virus. Thus, TCPs represent a good model to investigate single-cycle infection allowing analyses of antiviral compounds in the absence of secondary infection. As shown in Fig. 7, all 4 compounds efficiently inhibited the replication of the N17 replicon in cells infected with the VSV-G pseudotyped TCPs.

## Discussion

Using cell-based assays involving three different protocols, we show that most of our compounds inhibit HCV genome replication. Our initial data indicated that they had a moderate effect on the gt2a HCVcc entry. However, a further analysis using HCVpp, which is a well-established virus entry model, identified only Z431 as an HCV-specific entry inhibitor. Treatment of cells post-infection showed that most of the compounds inhibited viral genome replication by up to 95%. We excluded their possible effect on the translation of viral RNA by testing them during the translation phase of a replication-defective subgenomic viral replicon post-electroporation. That most of the compounds targeted viral genome replication was unequivocally confirmed using the viral sub-genomic replicon system. Interestingly, we found a significant difference in the antiviral activity of the compounds when tested in the stably established replicon cell lines as opposed to in cells that had been freshly electroporated with the replicon RNA. In the latter case, a stronger inhibitory activity was detected, indicating that these compounds likely act on *de novo* RNA synthesis thus affecting early stages of HCV replication. Our analysis of the effects of the selected compounds in the TCP system further reinforces this hypothesis.

Most of the compounds exhibited IC_50_ values in the micromolar scale and a good cell viability profile. The antiviral activity of the compound Z401 was in the nanomolar range. The Selectivity Index, expressed as the ratio of CC_50_ on IC_50,_ indicated that most of the compounds have high values (>10) and are therefore good candidates for further studies. Here, we selected four compounds for further studies based on an SI cut-off of 100. The selected compounds also inhibited replication of gt1b and gt3a replicon (with their IC50 values ranging from micromolar to nanomolar, respectively), confirming their pan-genotypic potential.

To further validate the mechanism of action of the selected compounds, we evaluated their effect on viral RNA levels in infected cells. We used well-established methods to quantitate both total RNA from infected cells and the positive-strand viral RNA from the released virion progeny. We used a cell line defective in the virus entry factor, CD81, thus excluding the possible effect of secondary infection and allowing analyses in a single cycle infection setting. As expected, compounds Z385, Z387, Z400 and Z401 efficiently inhibited the viral RNA and this inhibition corresponded to the reduced levels of viral genomic RNA (and hence virus release) in the cell medium. Moreover, in the HeF cells, in which CD81 expression was restored, a better inhibition was observed for all the compounds, probably due to their antiviral activity on secondary infection. These data indicated that our compounds act at an early stage of viral replication. To confirm this, we treated the electroporated cells for a short time (1–5 h) and then waited 24 or 48 h before evaluating viral replication. All the compounds showed viral inhibition, with Z401 being able to inhibit by up to 80%. However, a rebound in viral replication, especially after 48 h, was observed for all the compounds except Z401. Indeed, Z401 proved to be the most active compound blocking the replication of different HCV genotypes, 1b, 2a and 3a, with IC_50_ values of 2.2, 0.092 and 0.69 μM respectively. Overall, these data show that our compounds are highly efficient in blocking viral replication at an early stage and that their effects are stable. That compounds Z385, Z400 and Z401 do not act as direct inhibitors of HCV-encoded enzymes indicates that they may affect host factor(s) that are crucial for virus RNA replication. We are currently investigating this possibility.

SAR study has shown that the nature and position of R_1_ substituent have a significant effect on the inhibitory properties. Thus, unsubstituted compound Z436 (R_1_ = H) characterized with IC_50_ value of 3.62 μM and mediocre SI due to the relatively high cytotoxicity. Introduction of bromine atom at the *para*-position (compound Z263) led to some activity increase (IC_50_ 3.1 μM) and 5-fold SI increase. Replacement of the bromine atom with a cyano-group (compound Z434) or a phenyl residue (compound Z438) led to an increase in both the anti-HCV activity and cytotoxicity, which negatively affects the SI. At the same time, displacement of the bromine atom from 4- to 3-position (compound Z421) allowed to increase both the inhibitory activity (3 times) and SI (10 times) as compared to the unsubstituted Z436.

The nature of the spacer also plays a crucial role in the anti-HCV activity manifestation. Strong correlation is observed between spacer length and antiviral properties. While compounds Z397 and Z263, comprising four and five methylene units, respectively, show approximately the same level of anti-HCV activity, 6 methylene compound Z400 show an order of magnitude higher activity (IC_50_ 0.243 μM) and tremendously increased SI of 273. 8 Methylene compound Z401 show even higher level of inhibitory properties – 50% protective effect is evident at the 0.0924 μM concentration. However, concomitant increase of cytotoxicity led to some reduction in SI (244).

Apparently, the position of the oxygen atom in the spacer is also important. Shift of the oxygen from the aromatic moiety increase the activity twice (compound Z432). However, almost 3-fold increase in cytotoxicity is also observed. In turn, introduction of a second oxygen atom (compound Z422) results in a significant drop in the inhibitory properties and increased cytotoxicity. Replacement of the oxygen atom of Z436 with a methylene group in compound Z437 had little impact on activity.

Alkyl substituents in the uracil residue had practically no effect on the level of cytotoxicity, however, they influenced antiviral activity in a positive way. Thymine derivative Z376 is markedly more active than the parent Z263, while the 5-methyl-6-ethyluracil analogue (Z433) exceeded Z263 by two orders. In contrast, the introduction of an iodine atom in the 5-position of uracil residue (compound Z439) did not change the level of the inhibitory properties. At the same time, there was a marked increase in cytotoxicity.

Introduction of the R_3_ substituent also proved to have a favorable effect. Chlorine atom increased the activity 6-fold (compound Z385), a fluorine atom – nearly 5 times (compound Z414), methyl group − 10 times (Compound Z413) as compared to the unsubstituted compound Z263. However, for the latter two cases, there was a two-fold increase in cytotoxicity.

The study of influence of the nature of the linker fragment Y showed that optimal virus-inhibitory properties can be achieved with a methylene group (Z387). It provides the best activity and selectivity among the tested oxygen atom (Z263), carbonyl (Z430), or oxymethylene group (Z377).

Methylation of the acetanilide nitrogen atom increased the antiviral activity, but also led to a sharp increase in cytotoxicity (compound Z431) making impractical further modifications in this direction. Also it seems unfavorable to replace the long spacer with a methylene group (compound Z176). Due to the high cytotoxicity compound has a low SI.

## Materials and Methods

### Chemistry

General methods and techniques were applied to the synthesis of target compounds according to prior publications^20^. NMR spectra were obtained using Bruker Avance 400 spectrometer (400 MHz for ^1^H and 100 MHz for ^13^C) in DMSO-d_6_ or CDCl_3_ with tetramethylsilane as an internal standard. High-resolution mass spectra were measured on Bruker micrOTOF II instruments using electrospray ionization (HRESIMS). The measurements were run in positive ion mode (interface capillary voltage −4500 V) in a mass range from m/z 50 to m/z 3000 Da; external or internal calibration was performed with ESI Tuning Mix (Agilent Technologies). A syringe injection was used for solutions in MeCN (flow rate = 3 μL/min). N_2_ was applied as a dry gas; the interface temperature was set at 180 °C. The spectral data are presented in the on-line Supplementary file.

The HCV polymerase inhibitor, 2-C-methyladenosine^31^,^32^ was purchased from Sigma (UK).

### Cell Culture

Human epithelial kidney cells (HEK)-293T (ATCC CRL-1573), human hepatoma Huh7 cells (Nakabayashi *et al.*, 1982), Huh7.5-Sec14L2, Huh7- Lunet-CD81 cells and Huh7L-H/EF cells were grown in Dulbecco’s modified Eagle medium (Life Technologies) supplemented with 10% fetal calf serum, 2 mM L-glutamine, 100 U/ml penicillin, 100 μg/ml streptomycin and 0.1 M nonessential amino acids as described^27^,^28^. Huh7-J20 cells were propagated as above, but in the presence of 2 μg/ml puromycin (Sigma)^23^.

### Generation of cell culture infectious HCV (HCVcc) and retrovirus-based HCV and other pseudoparticles

The HCVcc used in this study was strain JFH-1 (kindly provided by T. Wakita as plasmid containing cDNA sequence^33^, and its cell-culture adaptive mutant JFH-1_DSGCSL_^29^. Infectious virus was generated and titrated as previously described^24^,^29^,^33^. HCVpp were generated in HEK-293T cells co-transfected with the retrovirus packaging vector pMLV gag-pol, the transfer vector pMLV-Luciferase and the HCV JFH-1 E1E2-expressing vector phCMV E1E2 as described previously^34^,^35^.

### Virus infection and drug screening assays

The Huh7-J20 reporter cell line, seeded into 96-well tissue culture dish, were infected with HCVcc in the presence or absence (i.e. DMSO control) of compounds and the levels of virus infectivity and replication were determined by measuring the secreted alkaline phosphatase (SEAP) activity in the culture medium at indicated time post-infection as described previously^23^. Antiviral screenings were performed using 3 different infection models. In the first one, cells were pre-treated with drugs for 1 h and then infected in the presence of the drugs. After 3 h, viral inoculum was replaced with fresh medium without drug and cells were incubated for 72 h. The second model was based on the first one but fresh drugs were present throughout the course of infection. In the third one, drugs were added at 3 h post-infection and cells incubated for 72 h. In all the three models, the antiviral activity was determined by measuring SEAP levels in the infected cell medium as described above.

For dose-response scales, Huh7-J20 were infected with HCVcc according to the second model described above. All the drugs were tested starting from the concentration of 30 μM with 3-fold dilutions. The antiviral activity was determined normalizing results to DMSO-treated cells and IC_50_ and CC_50_ values were calculated using a non-linear regression function with GraphPad Prism 6 software.

To determine the effect of compounds on virus entry, Huh7 cells seeded in 96-well plates were pre-treated for 1 h with compound and then infected with the HCVpp in the presence of the drug. DMSO was used as a vehicle control. After 3 h, the cells were washed and re-fed with fresh medium. At 72 h post-infection, HCVpp infectivity levels were determined by measuring luciferase activity.

### HCV Replicons

The monocistronic N17/JFH1 subgenomic replicon cDNA construct encoding the firefly luciferase reporter and the puromycin resistance marker (separated by the foot-and-mouth-disease virus [FMDV] 2a self-cleavage site) in the JFH1 ΔE1E2 background has been described previously^24^. N17/JFH1 replicon RNA was generated by *in vitro* transcription and electroporated into Huh7 cells as described^36^. At 3 days post-electroporation, the cells were cultured in the presence of 2 μg/ml puromycin and the surviving N17 replicon-expressing cells were pooled and established as a cell line designated Huh7-J17. For antiviral screening, Huh7-J17 cells seeded in 96-well plates were treated with the drugs or DMSO for 24, 48 or 72 h. Inhibition of virus RNA replication was assessed by measuring luciferase activity using the Bright-Glo Luciferase Assay system (Promega, UK) following removal of the medium and lysis of the cells.

The bicistronic gt2a subgenomic replicon constructs pUC JFH1-Luc and pUC JFH1-Luc/GND have been described previously^37^. The enhanced bicistronic gt3a subgenomic replicon S52-∆N, lacking of Neomycin resistance, has been described previously^26^,^27^. The Huh7 cell line stably harbouring the gt1b (isolate Con1) subgenomic I389/NS3-3′/LucUbiMeo/ET replicon has been previously reported^38^. These constructs lack the entire viral structural-encoding sequences and carry instead the firefly luciferase gene. The plasmid pUC JFH1-Luc/GND is identical to pUC JFH1-Luc except for the GND mutation in the viral NS5B RNA polymerase. The viral subgenomic RNA was transcribed *in vitro* from these constructs and electroporated into Huh7 cells. The cells were seeded into 96-well in the presence of drugs or DMSO and the virus RNA replication levels determined at indicated time post-electroporation by measuring luciferase activity as described above.

To evaluate the possible emergence of escape mutations, Huh7-J17 cells were cultured for 20 days in the presence of selected compounds. Every 3/4 days, cells were passaged in the presence of the drugs and the luciferase readings determined as relative light unit (RLU) from 20000 cells.

### HCV Trans-complemented particles (TCP)

Trans-complemented particles consisted of viral particles containing SGR-RNA as transgene, HCV Core protein that forms capsid and VSVg as glycoprotein expressed on the envelope. Briefly, 2 × 10^6^ Huh7-J17 cells, harboring N17 sub-genomic RNA, were transfected with 10 μg of phCMV-VSVg DNA using ViaFect (Promega) and incubated for 3 d. Supernatant-containing particles was then harvested and filtered through 0.45 μm filter. TCP were concentrated using PEG-it (Biosciences) by overnight incubation at 4 °C and then centrifuged at 1500 × g for 30 min. The pellet containing particles was resuspended in PBS, aliquoted and stored at −70 °C.

### RNA Quantification

Huh7 Lunet-CD81 cells and their control Huh7L- H/EF cells were electroporated with 5 μg of JFH-1 RNA, seeded with drugs in a 12-well tissue culture dish and incubated for 3 d. Cellular RNA was isolated using TRI Reagent (Sigma) following manufacturer’s protocol. RNA quantification from the released viral particles was performed as below following digestion of infected cell medium with RNAse A for 2 h and RNA extraction using the RNeasy kit (Qiagen). HCV RNA quantification was carried out by RT-qPCR using TaqMan probes as reported^39^. Briefly, 250 ng of total RNA were reverse transcribed to obtain cDNA in a 20 μl reaction using TaqMan Reverse Transcription Reagents (Life Technologies) with random hexamers, following manufacturer’s protocol. Reverse transcription was performed at 48 °C for 30 min, followed by 5 min at 85 °C. The qPCR was performed with 1.5 μl of cDNA in a 15 μl reaction mixture (TaqMan Fast Universal PCR Master Mix; Life Technologies) with 250 nM of Fw_primer (5′- TCCCGGGAGAGCCATAGTG-3′), 250 nm of Rev_primer (5′-TCCAAGAAAGGACCCAGTC-3′) and 250 nM of TaqMan MGB (minor-groove binding) probe labelled with 6-carboxyfluorescin (5′-FAM-TCTGCGGAACCGGTG-MGB-3′). Samples were placed in an ABI Fast 7500 instrument (Life Technologies) and were amplified using the following parameters: 95 °C for 5 minutes with cycling parameters set to 95 °C for 3 s and 60 °C for 30 s for 40 cycles. Absolute quantification was carried out using linear regression on a standard curve based on pJFH-1 serial dilution.

### Cell viability assay

Huh7, Huh7-J20 and Huh7-J17 cells were tested for viability in the same conditions described for antiviral assays. Cells grown in a 96-well tissue culture plate in the presence of the drugs or DMSO control were incubated with the WST-1 reagent (Roche) for 3 h as per the manufacturer’s protocol. Cell viability was obtained reading absorbance at 450 nm with PHERAstar (BMG Labtech).

### HCV enzyme activity assays

The helicase domain of the NS3 protein (Con1 isolate of genotype 1b, AF238799) was purified and its helicase and NTPase activity were measured as described earlier^40^. NS5BI21 protein (Con1 isolate of genotype 1b, AF238799) lacking C-terminal 21 amino acid residues was expressed in *E.coli* and purified as described earlier^41^. NS5BI21 from JFH1 isolate of genotype 2a (AB047639) was obtained, expressed and purified similarly. Their enzymatic activity was measured in a primer dependent and *de novo* assays as reported previously^41^,^42^. The detailed procedures are given in the Supplement.

### Statistical Analysis

All experiments were conducted at least in duplicate and repeated at least 3 times. Multiple-group comparison was performed by one-way analysis of variance. Data are presented as mean ± SEM. Statistical analysis was performed using GraphPad Prism 6 software. Statistical significance was defined as P < 0.05.

## Additional Information

**How to cite this article**: Magri, A. *et al.* Exploration of acetanilide derivatives of 1-(ω-phenoxyalkyl)uracils as novel inhibitors of Hepatitis C Virus replication. *Sci. Rep.* **6**, 29487; doi: 10.1038/srep29487 (2016).

## Supplementary Material

Supplementary Information

## Figures and Tables

**Table 1:** IC_50_, CC_50_ and SI values for all the compounds screened.

Compound	Spacer	R_1_	R_2_	R_3_	X	IC_50_, μM	CC_50_, μM	SI
Z263	―O(CH_2_)_5_―	4-Br	H	H	O	3.1	56.3	18.174
Z421	―O(CH_2_)_5_―	3-Br	H	H	O	1.16	44.6	38.548
Z434	―O(CH_2_)_5_―	4-CN	H	H	O	1.85	10.5	5.661
Z436	―O(CH_2_)_5_―	H	H	H	O	3.62	13.0	3.595
Z438	―O(CH_2_)_5_―	4-Ph	H	H	O	1.74	9.60	5.534
Z397	―O(CH_2_)_4_―	4-Br	H	H	O	2.95	61.1	20.715
Z400	―O(CH_2_)_6_―	4-Br	H	H	O	0.243	66.5	273.569
Z401	―O(CH_2_)_8_―	4-Br	H	H	O	0.0924	22.6	244.238
Z432	―CH_2_O(CH_2_)_4_―	4-Br	H	H	O	1.29	19.2	14.942
Z422	―CH_2_O(CH_2_)_2_OCH_2_―	4-Br	H	H	O	20	24.9	1.247
Z433	―CH_2_O(CH_2_)_2_OCH_2_―	4-Br	5-Et-6-Me	H	O	0.991	23.7	23.917
Z437	―(CH_2_)_6_―	H	H	H	O	2.93	14.7	5.029
Z376	―O(CH_2_)_5_―	4-Br	5-Me	H	O	2,18	69.2	31.761
Z439	―O(CH_2_)_5_―	4-Br	I	H	O	3.72	29.7	7.977
Z385	―O(CH_2_)_5_―	4-Br	H	Cl	O	0.585	59.2	101.145
Z413	―O(CH_2_)_5_―	4-Br	H	Me	O	0.315	27.4	87.166
Z414	―O(CH_2_)_5_―	4-Br	H	F	O	0.627	30.5	48.612
Z377	―O(CH_2_)_5_―	4-Br	H	H	OCH_2_	2.98	18.4	6.196
Z387	―O(CH_2_)_5_―	4-Br	H	H	CH_2_	0.712	132	185.482
Z430	―O(CH_2_)_5_―	4-Br	H	H	C = O	0.941	11.5	12.173
Z431	―O(CH_2_)_5_―	4-Br	H	H	O	0.5	2.67	5.348
Z176	―CH_2_―	H	H	H	O	1.34	17.6	13.174
2′-C-methyladenosine (Iro *et al.*^23^)						2	>30	>15

Huh7-J20 cells were infected following the second protocol as described in Methods. IC_50_, CC_50_ and SI values were calculated using GraphPad Prism 6 software.

**Table 2:** Inhibition of HCV gt1b and gt3a sub-genomic replicons by selected compounds.

Compound	IC_50_ (μM) Gt1b*	IC_50_ (μM) Gt3a**
Z385	6.9	0.66
Z387	23.3	0.94
Z401	2.2	0.69
Z400	12.6	1.1

^*^Huh7 cells stably harbouring the subgenomic I389/NS3-3′/LucUbiMeo/ET replicon were plated and compound dose response tests performed as described in Methods. IC_50_ values were calculated using GraphPad Prism 6 software.

^**^Huh7.5-SEC14L2 cells were electroporated with 2 μg of S52-WT-∆N RNA and plated and compound dose response tests performed as described in Methods. IC_50_ values were calculated using GraphPad Prism 6 software.
