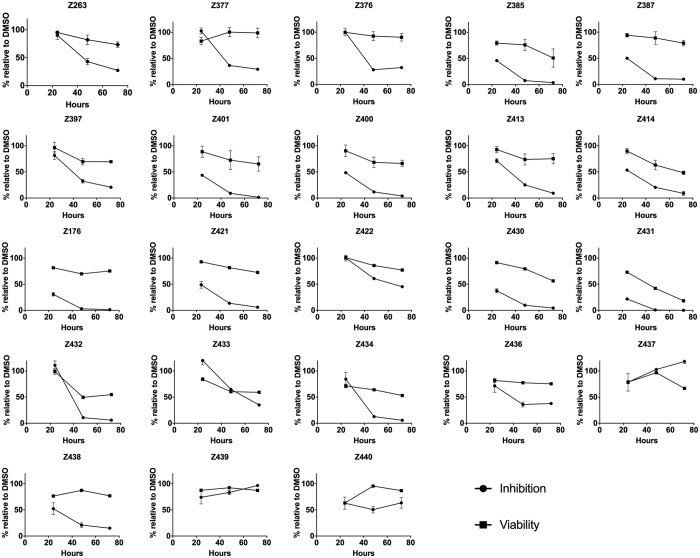# Corrigendum: Exploration of acetanilide derivatives of 1-(ω-phenoxyalkyl)uracils as novel inhibitors of Hepatitis C Virus replication

**DOI:** 10.1038/srep31529

**Published:** 2016-08-25

**Authors:** Andrea Magri, Alexander A. Ozerov, Vera L. Tunitskaya, Vladimir T. Valuev-Elliston, Ahmed Wahid, Mario Pirisi, Peter Simmonds, Alexander V. Ivanov, Mikhail S. Novikov, Arvind H. Patel

Scientific Reports 6: Article number: 29487; 10.1038/srep29487published online: 07 12 2016; updated: 08 25 2016

In this Article, Figure 3 is incorrect. The correct Figure 3 appears below as Figure 1. As a result, the accompanying legend should read:

“Antiviral activity on a replicon cell line. Huh-J17 cells expressing the N17/JFH1 subgenomic replicon were seeded in the presence of the drugs at the concentration of 10 μM each, or an equivalent amount of DMSO as a vehicle control, and then incubated at 37 °C for 24 h, 48 h or 72 h. The cells were then lysed and the luciferase activity (the level of which correlates directly with that of viral RNA replication) determined as described in Materials and Methods. In parallel, cell viability was measured at 72 h post-incubation. Data are presented as % activity (i.e. % inhibition of virus RNA replication or % cell viability) relative to that of DMSO-treated cells, which is represented as 100%”.

## Figures and Tables

**Figure 1:**