# Supplementary material for: Exploration of acetanilide derivatives of 1-(ω-phenoxyalkyl)uracils as novel inhibitors of Hepatitis C Virus replication
Source: Sci Rep. 2016 Jul 12;6:29487. doi: 10.1038/srep29487 (PMC4942610; doi:10.1038/srep29487)
Supplement: Supplementary Information [file srep29487-s1.pdf]

Exploration of acetanilide derivatives of 1-(ω-phenoxyalkyl)uracils as novel inhibitors of Hepatitis C Virus replication

Andrea Magri[1,4,5], Alexander A. Ozerov[2], Vera L. Tunitskaya[3], Vladimir T. Valuev-Elliston[3], Ahmed Wahid[1,6], Mario Pirisi[5], Peter Simmonds[4], Alexander V. Ivanov[3], Mikhail S. Novikov[2,#] and Arvind H. Patel[1#]

[1]MRC - University of Glasgow Centre for Virus Research, Glasgow, UK; [2]Department of Pharmaceutical & Toxicological Chemistry, Volgograd State Medical University, Volgograd, Russia; [3]Engelhardt Institute of Molecular Biology, Russian Academy of Science, Moscow, Russia; [4]Nuffield Department of Medicine, University of Oxford, Oxford, United Kingdom; [5]Department of Translational Medicine, Università del Piemonte Orientale, Novara, Italy; [6]Department of Biochemistry, Faculty of Pharmacy, Minia, University, Minia, Egypt.

#Arvind.Patel@glasgow.ac.uk and m-novikov1@mail.ru

Spectral data

2-[3-[5-(4-Bromophenoxy)pentyl]-2,6-dioxo-3,6-dihydropyrimidin-1(2H)-yl]-N-(4-phenoxyphenyl)acetamide (Z263). ^{1}H NMR (DMSO-d_6) (Fr 400 MHz): δ 1.40 (2H, qu, *J* = 7.8 Hz, CH_2), 1.66 (2H, qu, *J* = 7.2 Hz, CH_2), 1.72 (2H, qu, *J* = 7.3 Hz, CH_2), 3.76 (2H, t, *J* = 7.0 Hz, NCH_2), 3.93 (2H, t, *J* = 6.4 Hz, OCH_2), 4.61 (2H, s, $COCH_2$), 5.75 (1H, d, *J* = 7.8 Hz, H-5), 6.88 (2H, d, *J* = 8.9 Hz, H-2", H-6"), 6.96 (2H, d, *J* = 8.2 Hz, H-2"', H-6"'), 6.98 (2H, d, *J* = 8.4 Hz, H-2', H-6'), 7.09 (1H, dt, *J* = 7.3 and 0.9 Hz, H-4"'), 7.36 (2H, t, *J* = 7.6 Hz, H-3"', H-5"'), 7.40 (2H, d, *J* = 8.8 Hz, H-3', H-5'), 7.57 (2H, d, *J* = 8.9 Hz, H-3", H-5"), 7.77 (1H, d, *J* = 8.0 Hz, H-6), 10.27 (1H, s, NH). ^{13}C NMR (DMSO-d_6) (Fr 100 MHz): δ 22.3, 25.5, 28.1, 43.2, 48.5, 67.6, 100.0, 111.8, 116.7, 118.0, 119.5, 120.7, 123.0, 130.0, 132.1, 134.7, 144.6, 151.1, 151.8, 157.3, 157.9, 162.3, 165.1. HRMS: found *m/z* 578.1287; calcd for $C_{29}H_{28}BrN_3O_5$ $[M+H]^+$ 578.1285.

2-[3-[5-(3-Bromophenoxy)pentyl]-2,6-dioxo-3,6-dihydropyrimidin-1(2H)-yl]-N-(4-phenoxyphenyl)acetamide (Z421). ^{1}H NMR (DMSO-d_6) (Fr 400 MHz): δ 1.40 (2H, qu, *J* = 6.4 Hz, CH_2), 1.66 (2H, qu, *J* = 7.3 Hz, CH_2), 1.73 (2H, qu, *J* = 7.8 Hz, CH_2), 3.76 (2H, t, *J* = 7.3 Hz, CH_2N), 3.96 (2H, t, *J* = 6.4 Hz, CH_2O), 4.61 (2H, s, CH_2CO), 5.76 (1H, d, *J* = 7.8 Hz, H-5), 6.93 (1H, dd, *J* = 8.3 and 2.3 Hz, H-4'), 6-95-7.00 (4H, m, H-2", H-6", H-2"', H-6"'), 7.07-7.12 (3H, m, H-2', H-6', H-4"'), 7.20 (1H, t, *J* = 8.0 Hz, H-5'), 7.36 (2H, dt, *J* = 7.6 and 2.4 Hz, H-3"', H-5"'), 7.57 (2H, d, *J* = 9.0 Hz, H-3", H-5"), 7.77 (1H, d, *J* = 8.0 Hz, H-6), 10.27 (1H, s, NH). ^{13}C NMR (DMSO-d_6) (Fr 100 MHz): δ 22.6, 28.4, 40.7, 43.4, 48.8, 67.9, 100.3, 114.3, 117.5, 118.2, 119.8, 121.0, 122.4, 123.3, 123.6, 130.3, 131.5, 135.0, 144.8, 151.4, 152.1, 157.6, 160.0, 162.6, 165.4. HRMS: found *m/z* 578.1280; calcd for $C_{29}H_{28}BrN_3O_5$ $[M+H]^+$ 578.1285.

2-[3-[5-(4-Cyanophenoxy)pentyl]-2,6-dioxo-3,6-dihydropyrimidin-1(2H)-yl]-N-(4-phenoxyphenyl)acetamide (Z434). 1.41 (2H, qu, *J* = 8.5 Hz, CH_2), 1.67 (2H, qu, *J* = 7.4 Hz, CH_2), 1.76 (2H, qu, *J* = 7.6 Hz, CH_2), 3.76 (2H, t, *J* = 7.1 Hz, NCH_2), 4.05 (2H, t, *J* = 6.4 Hz, OCH_2), 4.60 (2H, s, $COCH_2$), 5.75 (1H, d, *J* = 8.0 Hz, H-5), 6.96 (2H, d, *J* = 8.8 Hz, H-2", H-6"), 6.98 (2H, d, *J* = 8.8 Hz, H-2"', H-6"'), 7.08 (2H, d, *J* = 9.1 Hz, H-2', H-6'), 7.09 (1H, t, *J* = 7.6 Hz, H-4"'), 7.36 (2H, dt, *J* = 7.4 and 1.2 Hz, H-3"', H-5"'), 7.57 (2H, d, *J* = 8.8 Hz, H-3', H-5'), 7.72 (2H, d, *J* = 8.8 Hz, H-3", H-5"), 7.77 (1H, d, *J* = 7.8 Hz, H-6), 10.27 (1H, s, NH). ^{13}C NMR (DMSO-d_6) (Fr 100 MHz): δ 22.5, 28.3, 28.4, 43.4, 48.7, 68.2, 100.3, 103.0, 115.8, 118.2, 119.5, 119.8, 121.0, 123.3,

130.3, 134.5, 135.0, 144.8, 151.4, 152.1, 157.6, 162.4, 162.5, 165.4. HRMS: found *m/z* 525.2126; calcd for $C_{30}H_{28}N_4O_5$ $[M+H]^+$ 525.2132.

2-[3-[5-(Phenoxy)pentyl]-2,6-dioxo-3,6-dihydropyrimidin-1(2H)-yl]-N-(4-phenoxyphenyl)-acetamide (Z436). ^1H NMR (DMSO-d_6) (Fr 400 MHz): δ 1.41 (2H, qu, *J* = 7.1 Hz, CH_2), 1.67 (2H, qu, *J* = 7.1 Hz, CH_2), 1.74 (2H, qu, *J* = 7.8 Hz, CH_2), 3.76 (2H, t, *J* = 7.0 Hz, NCH_2), 3.94 (2H, t, *J* = 6.3 Hz, OCH_2), 4.60 (2H, s, $COCH_2$), 5.75 (1H, d, *J* = 7.9 Hz, H-5), 6.87-6.91 (3H, m, H-4', H-2", H-6"), 6.95-6.99 (4H, m, H-2"', H-6"', H-2', H-6'), 7.10 (1H, dt, *J* = 7.3 and 1.0 Hz, H-4"'), 7.36 (2H, dt, *J* = 7.3 and 1.8 Hz, H-3"', H-5"'), 7.36 (2H, dt, *J* = 8.6 and 1.3 Hz, H-3', H-5'), 7.57 (2H, d, *J* = 9.0 Hz, H-3", H-5"), 7.78 (1H, d, *J* = 7.8 Hz, H-6), 10.26 (1H, s, NH). ^{13}C NMR (DMSO-d_6) (Fr 100 MHz): δ 22.7, 28.5, 28.6, 43.4, 48.8, 67.4, 100.3, 114.7, 118.2, 119.8, 120.7, 121.0, 123.3, 130.0, 130.3, 135.0, 144.9, 151.4, 152.1, 157.6, 158.9, 162.6, 165.4. HRMS: found *m/z* 500.2181; calcd for $C_{29}H_{29}N_3O_5$ $[M+H]^+$ 500.2180.

2-[3-[5-[(1,1'-biphenyl)-4-yloxy]pentyl]-2,6-dioxo-3,6-dihydropyrimidin-1(2H)-yl]-N-(4-phenoxyphenyl)acetamide (Z438). ^1H NMR (DMSO-d_6) (Fr 400 MHz): δ 1.43 (2H, qu, *J* = 8.1 Hz, CH_2), 1.68 (2H, qu, *J* = 7.1 Hz, CH_2), 1.76 (2H, qu, *J* = 7.6 Hz, CH_2), 3.78 (2H, t, *J* = 7.0 Hz, NCH_2), 3.99 (2H, t, *J* = 6.3 Hz, OCH_2), 4.61 (2H, s, $COCH_2$), 5.76 (1H, d, *J* = 8.0 Hz, H-5), 6.94-7.01 (6H, m, H-2', H-6', H-2", H-6", H-2"', H-6"'), 7.09 (1H, dt, *J* = 7.3 and 1.0 Hz, H-4"'), 7.29 (1H, dt, *J* = 7.3 and 1.0 Hz, Ph-H^4), 7.35 (2H, dt, *J* = 7.6 and 1.0 Hz, H-3"', H-5"'), 7.41 (2H, t, *J* = 7.3 Hz, Ph-H^3, Ph-H^5), 7.54-7.59 (6H, m, H-3', H-5', H-3", H-5", Ph-H^2, Ph-H^6), 7.79 (1H, d, *J* = 7.8 Hz, H-6), 10.27 (1H, s, NH). ^{13}C NMR (DMSO-d_6) (Fr 100 MHz): δ 22.7, 28.5, 28.6, 43.4, 45.4, 48.8, 67.6, 100.3, 115.2, 118.2, 119.8, 121.0, 123.3, 126.5, 127.0, 128.0, 129.2, 130.3, 132.7, 135.1, 140.2, 144.9, 151.4, 152.1, 157.7, 158.6, 162.6, 165.4. HRMS: found *m/z* 576.2487; calcd for $C_{35}H_{33}BrN_3O_5$ $[M+H]^+$ 576.2493.

2-[3-[4-(4-Bromophenoxy)butyl]-2,6-dioxo-3,6-dihydropyrimidin-1(2H)-yl]-N-(4-phenoxy-phenyl)acetamide (Z397). ^1H NMR (DMSO-d_6) (Fr 400 MHz): δ 1.72 (4H, m, CH_2CH_2), 3.80 (2H, t, *J* = 6.6 Hz, CH_2), 3.97 (2H, t, *J* = 5.9 Hz, CH_2), 4.60 (2H, s, CH_2CO), 5.76 (1H, d, *J* = 7.8 Hz, H-5), 6.90 (2H, d, *J* = 9.1 Hz, H-2", H-6"), 6.94-7.00 (4H, m, H-2"', H-6"', H-2', H-6'), 7.10 (1H, dt, *J* = 7.2 and 1.0 Hz, H-4"'), 7.36 (2H, t, 7.6 Hz, H-3"', H-5"'), 7.42 (2H, d, *J* = 9.0 Hz, H-3', H-5'), 7.57 (2H, d, *J* = 9.1 Hz, H-3", H-5"), 7.79 (1H, d, *J* = 8.1 Hz, H-6), 10.27 (1H, s, NH). ^{13}C NMR (DMSO-d_6) (Fr 100 MHz): δ 25.5, 25.8, 43.5, 48.7, 67.7, 100.4, 112.2, 117.1, 118.2, 119.8, 121.0, 123.3, 130.3, 132.4, 135.0, 144.8, 151.4, 152.1, 157.6, 158.2, 162.5, 165.4. HRMS: found *m/z* 564.1127; calcd for $C_{28}H_{26}BrN_3O_5$ $[M+H]^+$ 564.1129.

2-[3-[6-(4-Bromophenoxy)hexyl]-2,6-dioxo-3,6-dihydropyrimidin-1(2H)-yl]-N-(4-phenox-yphenyl)acetamide (Z400). ^1H NMR ($CDCl_3$) (Fr 400 MHz): δ 1.42 (2H, qu, *J* = 7.6 Hz, CH_2), 1.52 (2H, qu, *J* = 7.1 Hz, CH_2), 1.73-1.79 (4H, m, CH_2CH_2), 3.76 (2H, t, *J* = 7.8 Hz, NCH_2), 3.93 (2H, t, *J* = 6.3 Hz, OCH_2), 4.77 (2H, s, $COCH_2$), 5.77 (1H, d, *J* = 7.8 Hz, H-5), 6.76 (2H, d, *J* = 8.8 Hz, H-2", H-6"), 6.93 (2H, d, *J* = 8.8 Hz, H-2"', H-6"'), 6.97 (2H, d, *J* = 8.3 Hz, H-2', H-6'), 7.07 (1H, dt, *J* = 7.1 and 0.9 Hz, H-4"'), 7.14 (1H, d, *J* = 7.9 Hz, H-6), 7.28-7.37 (4H, m, H-3"', H-5"', H-3', H-5'), 7.44 (2H, d, *J* = 8.8 Hz, H-3", H-5"), 7.95 (1H, s, NH). ^{13}C NMR ($CDCl_3$) (Fr 100 MHz): δ 25.4, 25.9, 28.7, 28.8, 49.6, 67.9, 101.2, 116.3, 118.4, 119.3, 122.9, 129.5, 132.1, 142.5, 151.3, 162.6. HRMS: found *m/z* 592.1437; calcd for $C_{30}H_{30}BrN_3O_5$ $[M+H]^+$ 592.1442.

2-[3-[8-(4-Bromophenoxy)octyl]-2,6-dioxo-3,6-dihydropyrimidin-1(2H)-yl]-N-(4-phenoxyphenyl)acetamide (Z401). ^{1}H NMR ($CDCl_3$) (Fr 400 MHz): δ 1.34 (6H, m, CH_2 × 6), 1.42 (2H, qu, *J* = 7.6 Hz, CH_2), 1.71 (2H, qu, *J* = 7.1 Hz, CH_2), 1.75 (2H, qu, *J* = 8.1 Hz, CH_2), 3.73 (2H, t, *J* = 7.4 Hz, NCH_2), 3.90 (2H, t, *J* = 6.6 Hz, OCH_2), 4.81 (2H, s, $COCH_2$), 5.80 (1H, d, *J* = 7.8 Hz, H-5), 6.77 (2H, d, *J* = 9.0 Hz, H-2", H-6"), 6.88 (2H, d, *J* = 8.8 Hz, H-2"', H-6"'), 6.94 (2H, d, *J* = 7.8 Hz, H-2', H-6'), 7.06 (1H, t, *J* = 7.3 Hz, H-4"'), 7.17 (1H, d, *J* = 7.8 Hz, H-6), 7.30-7.37 (4H, m, H-3"', H-5"', H-3', H-5'), 7.43 (2H, d, *J* = 8.8 Hz, H-3", H-5"), 8.52 (1H, s, NH). ^{13}C NMR ($CDCl_3$) (Fr 100 MHz): δ 25.7, 26.2, 28.8, 28.91, 28.94, 29.0, 44.2, 49.9, 68.0, 76.6, 76.9, 77.3, 101.2, 112.4, 116.2, 118.2, 119.3, 121.2, 122.8, 129.6, 132.1, 133.3, 143.0, 151.3, 153.1, 156.9, 158.1, 162.9, 164.8. HRMS: found *m/z* 620.1749; calcd for $C_{32}H_{34}BrN_3O_5$ $[M+H]^+$ 620.1755.

2-[3-[4-(4-Bromobenzyloxy)butyl]-2,6-dioxo-3,6-dihydropyrimidin-1(2H)-yl]-N-(4-phenoxyphenyl)acetamide (Z432). ^{1}H NMR (DMSO-*d*$_6$) (Fr 400 MHz): δ 1.54 (2H, qu, *J* = 6.3 Hz, CH_2), 1.67 (2H, qu, *J* = 7.4 Hz, CH_2), 3.44 (2H, t, *J* = 6.1 Hz, CH_2), 3.75 (2H, t, *J* = 7.1 Hz, CH_2), 4.42 (2H, s, $ArCH_2$), 4.60 (2H, s, CH_2CO), 5.75 (1H, d, *J* = 7.9 Hz, H-5), 6.96 (2H, d, *J* = 8.1 Hz, H-2"', H-6"'), 6.98 (2H, d, *J* = 9.1 Hz, H-2", H-6"), 7.09 (1H, t, *J* = 7.3 Hz, H-4"'), 7.27 (2H, d, *J* = 8.3 Hz, H-2', H-6'), 7.36 (2H, dt, *J* = 7.6 and 2.0 Hz, H-3"', H-5"'), 7.52 (2H, d, *J* = 8.3 Hz, H-3', H-5'), 7.57 (2H, d, *J* = 7.1 Hz, H-3", H-5"), 7.76 (1H, d, *J* = 7.8 Hz, H-6), 10.27 (1H, s, NH).^{13}C NMR (DMSO-*d*$_6$) (Fr 100 MHz): δ 25.7, 26.4, 43.4, 45.4, 48.8, 69.6, 71.3, 100.3, 118.2, 119.8, 120.7, 121.0, 123.3, 129.9, 130.3, 131.5, 135.0, 138.4, 144.8, 151.4, 152.1, 157.6, 162.6, 165.4. HRMS: found *m/z* 578.1287; calcd for $C_{29}H_{28}BrN_3O_5$ $[M+H]^+$ 578.1285.

2-[3-[[2-(4-Bromobenzyloxy)ethoxy]methyl]-2,6-dioxo-3,6-dihydropyrimidin-1(2H)-yl]-N-(4-phenoxyphenyl)acetamide (Z422). ^{1}H NMR (DMSO-*d*$_6$) (Fr 400 MHz): δ 3.55 (2H, t, *J* = 5.9 Hz, CH_2), 3.68 (2H, t, *J* = 4.9 Hz, CH_2), 4.45 (2H, s, $ArCH_2$), 4.61 (2H, s, CH_2CO), 5.19 (2H, s, NCH_2O), 5.82 (1H, d, *J* = 7.8 Hz, H-5), 6.96 (2H, d, *J* = 9.0 Hz, H-2"', H-6"'), 6.98 (2H, d, *J* = 9.0 Hz, H-2", H-6"), 7.09 (1H, t, *J* = 7.3 Hz, H-4"'), 7.27 (2H, d, *J* = 8.3 Hz, H-2', H-6'), 7.36 (2H, dt, *J* = 8.6 and 1.0 Hz, H-3"', H-5"'), 7.52 (2H, d, *J* = 8.3 Hz, H-3', H-5'), 7.56 (2H, d, *J* = 7.1 Hz, H-3", H-5"), 7.83 (1H, d, *J* = 7.9 Hz, H-6), 10.30 (1H, s, NH).^{13}C NMR (DMSO-*d*$_6$) (Fr 100 MHz): δ 43.4, 68.6, 69.2, 71.5, 78.0, 101.1, 118.3, 119.8, 120.7, 121.0, 123.3, 129.9, 130.3, 131.5, 135.0, 138.2, 144.2, 151.6, 152.1, 157.6, 162.4, 165.3. HRMS: found *m/z* 580.1073; calcd for $C_{28}H_{26}BrN_3O_6$ $[M+H]^+$ 580.1078.

1-[[2-(4-Bromobenzyloxy)ehtoxy]methyl]-3-[2-(4-phenoxyphenylamino)-2-oxoethyl]-5-ethyl-6-methyluracil (Z433). ^{1}H NMR (DMSO-*d*$_6$) (Fr 400 MHz): δ 0.95 (3H, t, *J* = 7.6 Hz, CH_3), 2.35 (3H, s, CH_3), 2.37 (2H, q, *J* = 7.3 Hz, CH_2), 3.56 (2H, t, *J* = 6.4 Hz, CH_2), 3.68 (2H, t, *J* = 6.3 Hz, CH_2), 4.45 (2H, s, $ArCH_2$), 4.64 (2H, s, CH_2CO), 5.38 (2H, s, NCH_2O), 6.96 (2H, d, *J* = 8.5 Hz, H-2"', H-6"'), 6.98 (2H, d, *J* = 9.1 Hz, H-2", H-6"), 7.09 (1H, t, *J* = 7.4 Hz, H-4"'), 7.26 (2H, d, *J* = 8.3 Hz, H-2', H-6'), 7.36 (2H, dt, *J* = 8.5 and 1.4 Hz, H-3"', H-5"'), 7.51 (2H, d, *J* = 8.4 Hz, H-3', H-5'), 7.57 (2H, d, *J* = 9.0 Hz, H-3", H-5"), 10.28 (1H, s, NH). ^{13}C NMR (DMSO-*d*$_6$) (Fr 100 MHz): δ 13.8, 14.8, 19.1, 44.0, 68.1, 69.2, 71.4, 74.1, 112.8, 118.2, 119.8, 120.7, 121.0, 123.3, 129.8, 130.3, 131.4, 135.1, 138.2, 148.0, 151.9, 152.1, 157.7, 161.9. 165.4. HRMS: found *m/z* 644.1346; calcd for $C_{31}H_{32}BrN_3O_6$ $[M+Na]^+$ 644.1367.

2-[2,6-Dioxo-3-(6-phenylhexyl)-3,6-dihydropyrimidin-1(2H)-yl]-N-(4-phenoxyphenyl)acetamide (Z437). ^{1}H NMR (Fr 400 MHz, DMSO-d$_6$): δ 1.22-1.37 (4H, m, CH_2), 1.49-1.65 (4H, m, CH_2), 2.55 (2H, t, J = 7.4 Hz, CH_2), 3.72 (2H, t, J = 7.1 Hz, CH_2), 4.61 (2H, s, CH_2), 5.74 (1H, d, J = 7.8 Hz, Ura-H-5), 6.93-7.00 (4H, m, H-3', H-5', H-2", H-6"), 7.07-7.12 (1H, m, H-4"), 7.13-7.19 (3H, m, H-4, H-2, H-6), 7.21-7.27 (2H, m, H-3, H-5), 7.33-7.39 (2H, m, H-2', H-6'),

7.54-7.60 (2H, m, H-3”, H-5”), 7.75 (1H, d, J = 7.8 Hz, Ura-H-6), 10.27 (1H, s, NH); ^{13}C NMR (100 MHz, DMSO-d$_6$): δ 25.9, 28.5, 28.6, 31.1, 35.3, 43.4, 48.9, 100.2, 118.2, 119.8, 121.0, 123.3, 125.9, 128.5, 128.5, 130.3, 135.0, 142.5, 144.8, 151.4, 152.1, 157.6, 162.6, 165.4; HRMS: found m/z 498.2386; calcd for $C_{30}H_{31}N_3O_4$ $[M+H]^+$ 498.2387.

2-[3-[5-(4-Bromophenoxy]pentyl]-5-methyl-2,6-dioxo-3,6-dihydropyrimidin-1(2H)-yl]-N-(4-phenoxyphenyl)acetamide (Z376). ^{1}H NMR (DMSO-*d*$_6$) (Fr 400 MHz): δ 1.39 (2H, qu, *J* = 6.9 Hz, CH_2), 1.66 (2H, qu, *J* = 7.1 Hz, CH_2), 1.72 (2H, qu, *J* = 7.1 Hz, CH_2), 1.83 (3H, s, CH_3), 3.72 (2H, t, *J* = 6.8 Hz, NCH_2), 3.92 (2H, t, *J* = 6.3 Hz, OCH_2), 4.63 (2H, s, $COCH_2$), 6.87 (2H, d, *J* = 8.8 Hz, H-2”, H-6”), 6.96 (2H, d, *J* = 8.1 Hz, H-2”’, H-6”’), 6.98 (2H, d, *J* = 8.5 Hz, H-2’, H-6’), 7.09 (1H, t, *J* = 7.3 Hz, H-4”’), 7.35 (2H, t, *J* = 7.8 Hz, H-3”’, H-5”’), 7.39 (2H, d, *J* = 8.8 Hz, H-3’, H-5’), 7.57 (2H, d, *J* = 8.8 Hz, H-3”, H-5”), 7.66 (1H, s, H-6), 10.28 (1H, s, NH). ^{13}C NMR (DMSO-*d*$_6$) (Fr 100 MHz): δ 12.9, 22.6, 28.4, 43.7, 48.5, 67.8, 107.9, 112.0, 117.0, 118.2, 119.8, 120.9, 123.3, 130.3, 132.4, 135.1, 140.7, 151.2, 152.1, 157.6, 158.2, 163.3, 165.5. HRMS: found *m/z* 592.1437; calcd for $C_{30}H_{30}BrN_3O_5$ $[M+H]^+$ 592.1442.

2-[3-[5-(4-Bromophenoxy]pentyl]-5-iodo-2,6-dioxo-3,6-dihydropyrimidin-1(2H)-yl]-N-(4-phenoxyphenyl)acetamide (Z439). ^{1}H NMR (DMSO-*d*$_6$) (Fr 400 MHz): δ 1.39 (2H, qu, *J* = 8.1 Hz, CH_2), 1.66 (2H, qu, *J* = 7.1 Hz, CH_2), 1.72 (2H, qu, *J* = 7.9 Hz, CH_2), 3.78 (2H, t, *J* = 7.0 Hz, NCH_2), 3.93 (2H, t, *J* = 6.6 Hz, OCH_2), 4.65 (2H, s, $COCH_2$), 6.88 (2H, d, *J* = 9.0 Hz, H-2”, H-6”), 6.96 (2H, d, *J* = 8.1 Hz, H-2”’, H-6”’), 6.98 (2H, d, *J* = 8.8 Hz, H-2’, H-6’), 7.10 (1H, dt, *J* = 7.3 and 0.9 Hz, H-4”’), 7.36 (2H, dt, *J* = 7.5 and 1.0 Hz, H-3”’, H-5”’), 7.40 (2H, d, *J* = 9.0 Hz, H-3’, H-5’), 7.56 (2H, d, *J* = 9.0 Hz, H-3”, H-5”), 8.37 (1H, s, H-6), 10.28 (1H, s, NH). ^{13}C NMR (DMSO-*d*$_6$) (Fr 100 MHz): δ 22.5, 28.4, 28.5, 45.0, 49.1, 67.2, 67.9, 112.1, 117.1, 118.3, 119.8, 121.0, 123.4, 130.3, 132.4, 134.9, 149.1, 151.0, 152.2, 157.6, 158.2, 160.2, 165.2. HRMS: found *m/z* 704.0248; calcd for $C_{29}H_{27}BrIN_3O_5$ $[M+H]^+$ 704.0252.

2-[3-[5-(4-Bromophenoxy)pentyl]-2,6-dioxo-3,6-dihydropyrimidin-1(2H)-yl]-N-[4-(4-chloro-phenoxy)phenyl]acetamide (Z385). ^{1}H NMR (DMSO-*d*$_6$) (Fr 400 MHz): δ 1.38 (2H, qu, *J* = 7.8 Hz, CH_2), 1.65 (2H, qu, *J* = 7.4 Hz, CH_2), 1.71 (2H, qu, *J* = 7.6 Hz, CH_2), 3.74 (2H, t, *J* = 7.1 Hz, NCH_2), 3.91 (2H, t, *J* = 6.3 Hz, OCH_2), 4.60 (2H, s, $COCH_2$), 5.75 (1H, d, *J* = 7.8 Hz, H-5), 6.98 (2H, d, *J* = 9.0 Hz, H-3’, H-5’), 6.97 (2H, d, *J* = 9.0 Hz, H-3”, H-5”), 6.99 (2H, d, *J* = 9.0 Hz, H-2”’, H-6”’), 7.38 (4H, d, *J* = 8.8 Hz, H-2’, H-6’, H-2”, H-6”), 7.57 (2H, d, *J* = 9.1 Hz, H-2”’, H-6”’), 7.75 (1H, d, *J* = 8.1 Hz, H-6), 10.32 (1H, s, NH). ^{13}C NMR (DMSO-*d*$_6$) (Fr 100 MHz): δ 22.6, 28.4, 43.4, 48.8, 100.3, 112.0, 117.0, 119.8, 120.0, 121.0, 127.0, 130.1, 132.4, 135.3, 144.9, 151.4, 151.7, 156.6, 158.2, 162.6, 165.5. HRMS: found *m/z* 634.0706; calcd for $C_{29}H_{27}BrClN_3O_5$ $[M+Na]^+$ 634.0715.

2-[3-[5-(4-Bromophenoxy)pentyl]-2,6-dioxo-3,6-dihydropyrimidin-1(2H)-yl]-N-[4-(4-methyl-phenoxy)phenyl]acetamide (Z413). ^{1}H NMR (DMSO-*d*$_6$) (Fr 400 MHz): δ 1.39 (2H, qu, *J* = 7.8 Hz, CH_2), 1.65 (2H, qu, *J* = 7.3 Hz, CH_2), 1.72 (2H, qu, *J* = 6.6 Hz, CH_2), 2.26 (3H, s, CH_3), 3.75 (2H, t, *J* = 6.8 Hz, NCH_2), 3.92 (2H, t, *J* = 6.2 Hz, OCH_2), 4.59 (2H, s, $COCH_2$), 5.75 (1H, d, *J* = 7.8 Hz, H-5), 6.85-6.88 (4H, m, H-3’, H-5’, H-2”’, H-6”’), 6.93 (2H, d, *J* = 8.8 Hz, H-2”, H-6”), 7.16 (2H, d, *J* = 8.1 Hz, H-3”’, H-5”’), 7.39 (2H, d, *J* = 8.8 Hz, H-2’, H-6’), 7.53 (2H, d, *J* = 9.0 Hz, H-3”, H-5”), 7.76 (1H, d, *J* = 8.1 Hz, H-6), 10.25 (1H, s, NH). ^{13}C NMR (DMSO-*d*$_6$) (Fr 100 MHz): δ 20.5, 22.6, 28.4, 43.4, 48.8, 67.9, 100.3, 112.0, 117.0, 118.6, 119.2, 121.0, 130.6, 132.4, 132.5,

134.6, 144.8, 151.4, 152.8, 155.1, 158.2, 162.6, 165.4. HRMS: found *m/z* 614.1246; calcd for $C_{30}H_{30}BrN_3O_5$ $[M+Na]^+$ 614.1261.

2-[3-[5-(4-Bromophenoxy)pentyl]-2,6-dioxo-3,6-dihydropyrimidin-1(2H)-yl]-N-[4-(4-fluoro-phenoxy)phenyl]acetamide (Z414). ^1H NMR (DMSO-*d*$_6$) (Fr 400 MHz): δ 1.39 (2H, qu, *J* = 7.3 Hz, CH_2), 1.65 (2H, qu, *J* = 6.8 Hz, CH_2), 1.71 (2H, qu, *J* = 7.1 Hz, CH_2), 3.75 (2H, t, *J* = 7.6 Hz, NCH_2), 3.92 (2H, t, *J* = 5.9 Hz, OCH_2), 4.60 (2H, s, $COCH_2$), 5.75 (1H, d, *J* = 7.8 Hz, H-5), 6.87 (2H, d, *J* = 8.8 Hz, H-2", H-6"), 6.95 (2H, d, *J* = 8.6 Hz, H-2', H-6'), 7.00-7.02 (2H, m, H-2"', H-6"'), 7.16-7.21 (2H, m, H-3"', H-5"'), 7.39 (2H, d, *J* = 8.8 Hz, H-3', H-5'), 7.55 (2H, d, *J* = 8.8 Hz, H-3", H-5"), 7.78 (1H, d, *J* = 7.8 Hz, H-6), 10.27 (1H, s, NH). ^{13}C NMR (DMSO-*d*$_6$) (Fr 100 MHz): δ 14.3, 22.4, 22.6, 31.3, 43.4, 48.8, 67.9, 100.3, 112.0, 116.7, 116.9, 117.0, 119.3, 120.2, 120.3, 121.0, 132.4, 134.9, 144.8, 151.4, 152.7, 153.5, 157.1, 158.2, 159.5, 162.6, 165.4. HRMS: found *m/z* 618.0996; calcd for $C_{29}H_{27}BrFN_3O_5$ $[M+Na]^+$ 618.1010.

2-[3-[5-(4-Bromophenoxy)pentyl]-2,6-dioxo-3,6-dihydropyrimidin-1(2H)-yl]-N-(4-benzyloxy-phenyl)acetamide (Z377). ^1H NMR (DMSO-*d*$_6$) (Fr 400 MHz): δ 1.38 (2H, qu, *J* = 7.8 Hz, CH_2), 1.64 (2H, qu, *J* = 7.4 Hz, CH_2), 1.71 (2H, qu, *J* = 7.5 Hz, CH_2), 3.74 (2H, t, *J* = 7.1 Hz, NCH_2), 3.91 (2H, t, *J* = 6.4 Hz, OCH_2), 4.58 (2H, s, $COCH_2$), 5.05 (2H, s, OCH_2Ph), 5.74 (1H, d, *J* = 8.1 Hz, H-5), 6.87 (2H, d, *J* = 9.1 Hz, H-3', H-5'), 6.94 (2H, d, *J* = 8.2 Hz, H-2"', H-6"'), 7.31 (1H, t, *J* = 7.1 Hz, H-4"), 7.35-7.46 (8H, aromatic H), 7.73 (1H, d, *J* = 8.0 Hz, H-6), 10.13 (1H, s, NH). ^{13}C NMR (DMSO-*d*$_6$) (Fr 100 MHz): δ 22.6, 28.4, 43.4, 48.8, 67.9, 69.7, 100.3, 112.0, 115.2, 117.0, 120.8, 128.0, 128.1, 128.7, 132.4, 137.4, 144.8, 151.4, 154.6, 158.2, 162.6, 165.1. HRMS: found *m/z* 592.1437; calcd for $C_{30}H_{30}BrN_3O_5$ $[M+H]^+$ 592.1442.

2-[3-[5-(4-Bromophenoxy)pentyl]-2,6-dioxo-3,6-dihydropyrimidin-1(2H)-yl]-N-(4-benzyl-phenyl)acetamide (Z387). ^1H NMR (DMSO-*d*$_6$) (Fr 400 MHz): δ 1.39 (2H, qu, *J* = 6.9 Hz, CH_2), 1.65 (2H, qu, *J* = 7.1 Hz, CH_2), 1.72 (2H, qu, *J* = 7.1 Hz, CH_2), 3.75 (2H, t, *J* = 6.9 Hz, NCH_2), 3.87 (2H, s, $PhCH_2$), 3.92 (2H, t, *J* = 6.4 Hz, OCH_2), 4.59 (2H, s, $COCH_2$), 5.75 (1H, d, *J* = 7.8 Hz, H-5), 6.88 (2H, d, *J* = 9.1 Hz, H-2", H-6"), 7.13-7.20 (5H, m, aromatic H), 7.25-7.28 (2H, m, aromatic H), 7.40 (2H, d, *J* = 9.0 Hz, H-3", H-5"), 7.46 (2H, d, *J* = 8.5 Hz, H-3', H-5'), 7.77 (1H, d, *J* = 7.8 Hz, H-6), 10.20 (1H, s, NH). ^{13}C NMR (DMSO-*d*$_6$) (Fr 100 MHz): δ 22.6, 28.4, 43.4, 48.7, 67.8, 100.28, 112.1, 117.0, 119.4, 126.2, 128.7, 128.9, 129.3, 132.4, 136.5, 137.1, 141.7, 144.8, 151.4, 158.2, 162.6, 165.4. HRMS: found *m/z* 576.1487; calcd for $C_{30}H_{30}BrN_3O_4$ $[M+H]^+$ 576.1492.

2-[3-[5-(4-Bromophenoxy)pentyl]-2,6-dioxo-3,6-dihydropyrimidin-1(2H)-yl]-N-(4-benzoyl-phenyl)acetamide (Z430). ^1H NMR (DMSO-*d*$_6$) (Fr 400 MHz): δ 1.40 (2H, qu, *J* = 8.1 Hz, CH_2), 1.66 (2H, qu, *J* = 7.1 Hz, CH_2), 1.72 (2H, qu, *J* = 7.8 Hz, CH_2), 3.76 (2H, t, *J* = 7.1 Hz, NCH_2), 3.92 (2H, t, *J* = 6.3 Hz, OCH_2), 4.67 (2H, s, $COCH_2$), 5.77 (1H, d, *J* = 7.8 Hz, H-5), 6.87 (2H, d, *J* = 9.1 Hz, H-2', H-6'), 7.39 (2H, d, *J* = 9.0 Hz, H-3', H-5'), 7.54 (2H, t, *J* = 7.3 Hz, H-3"', H-5"'), 7.65 (1H, dt, *J* = 7.6 and 1.5 Hz, H-4"'), 7.69-7.75 (6H, m, H-2", H-6", H-3", H-5", H-2"', H-6"'), 7.79 (1H, d, *J* = 8.0 Hz, H-6), 10.67 (1H, s, NH). ^{13}C NMR (DMSO-*d*$_6$) (Fr 100 MHz): δ 22.6, 28.4, 43.7, 48.8, 67.8, 100.3, 112.1, 117.0, 118.6, 128.8, 129.7, 131.6, 131.8, 132.4, 132.6, 137.9, 143.2, 144.9, 151.4, 158.2, 162.5, 166.3, 194.9. HRMS: found *m/z* 590.1283; calcd for $C_{30}H_{28}BrN_3O_5$ $[M+H]^+$ 590.1285.

4-[3-[5-(4-Bromophenoxy)pentyl]-2,6-dioxo-3,6-dihydropyrimidin-1(2*H*)-yl]-*N*-methyl-*N*-(4-phenoxyphenyl)acetamide (Z431). ^1H NMR (DMSO-*d*$_6$) (Fr 400 MHz): δ 1.37 (2H, qu, *J* = 7.8 Hz, CH_2), 1.63 (2H, qu, *J* = 7.1 Hz, CH_2), 1.71 (2H, qu, *J* = 7.1 Hz, CH_2), 3.14 (3H, s, CH_3), 3.72 (2H, t, *J* = 7.0 Hz, NCH_2), 3.93 (2H, t, *J* = 6.3 Hz, OCH_2), 4.26 (2H, s, $COCH_2$), 5.70 (1H, d, *J* =

7.8 Hz, H-5), 6.88 (2H, d, *J* = 8.5 Hz, H-2”, H-6”), 7.06-7.11 (4H, m, H-2’, H-6’, H-2”’, H-6”’), 7.18 (1H, t, *J* = 7.5 Hz, H-4”’), 7.39-7.43 (6H, m, H-3’, H-5’, H-3”, H-5”, H-3”’, H-5”’), 7.73 (1H, d, *J* = 7.8 Hz, H-6). ^{13}C NMR (DMSO-d_6) (Fr 100 MHz): δ 22.6, 28.4, 37.4, 42.3, 48.7, 60.1, 67.8, 100.2, 112.1, 117.0, 119.4, 119.8, 124.3, 129.5, 130.5, 132.4, 133.0, 137.8, 144.7, 151.2, 156.7, 158.2, 162.4, 166.2. HRMS: found *m/z* 592.1439; calcd for $C_{30}H_{30}BrN_3O_5$ $[M+H]^+$ 592.1442.

2-(3-Benzyl-2,6-dioxo-3,6-dihydropyrimidin-1(2*H*)-yl)-*N*-(4-phenoxyphenyl)acetamide (Z176). ^{1}H NMR (DMSO-d_6) (Fr 400 MHz): δ 4.65 (2H, s, $COCH_2$), 4.97 (2H, s, $ArCH_2$), 5.82 (1H, d, *J* = 7.9 Hz, H-5), 6.97 (2H, d, *J* = 8.1 Hz, H-2”’, H-6”’), 7.00 (2H, d, *J* = 9.0 Hz, H-2”, H-6”), 7.09 (1H, t, *J* = 7.3 Hz, H-4”’), 7.28-7.38 (7H, m, C_6H_5, H-3”’, H-5”’), 7.60 (2H, d, *J* = 9.0 Hz, H-3”, H-5”), 7.88 (1H, d, *J* = 7.8 Hz, H-6), 10.32 (1H, s, NH).^{13}C NMR (DMSO-d_6) (Fr 100 MHz): δ 42.9, 51.0, 100.1, 117.5, 119.1, 120.3, 122.6, 127.1, 127.4, 128.3, 129.5, 134.3, 136.1, 144.1, 150.8, 151.4, 156.9, 161.8, 164.7. HRMS: found *m/z* 450.1411; calcd for $C_{25}H_{21}BrN_3O_4$ $[M+Na]^+$ 450.1424.

1-[5-(4-Bromophenoxy)pentyl]uracil (Z149). ^{1}H NMR (DMSO-d_6): δ 1.39 (2H, qu, *J* = 8.3 Hz, CH_2), 1.62 (2H, qu, *J* = 7.2 Hz, CH_2), 1.71 (2H, qu, *J* = 7.2 Hz, CH_2), 3.66 (2H, t, *J* = 7.2 Hz, NCH_2), 3.92 (2H, t, *J* = 6.3 Hz, OCH_2), 5.55 (1H, dd, *J* = 7.8 and 2.2 Hz, H-5), 6.87 (2H, d, *J* = 9 Hz, H-3’, H-5’), 7.41 (2H, d, *J* = 8.9 Hz, H-2’, H-6’), 7.64 (1H, d, *J* = 7.8 Hz, H-6), 11.23 (1H, s, N^3H). ^{13}C NMR (DMSO-d_6): δ 22.8, 28.6, 47.8, 68.0, 101.3, 112.2, 117.1, 132.5, 146.2, 151.4, 158.3, 164.3. HRMS: found m/z 375.0310, calcd for $C_{15}H_{17}BrN_2O_3$ [M+Na]+ m/z 375.0315.

Enzymatic activity assays

The helicase domain of the NS3 protein (Con1 isolate of genotype 1b, AF238799) was purified as described earlier 1. Its helicase and NTPase activity was measured as described herein. The compounds dissolved in DMSO were added to the final concentration of 50 μM, and an equal amount of DMSO as a vehicle was added to the control reaction (10% final concentration).

NS5BΔ21 (Con1 isolate of genotype 1b, AF238799) lacking 21 amino acid residues at C-terminus of the polypeptide chain was expressed in E.coli and purified as described earlier 2. Its enzymatic activity was measured in a primer dependent assay based on polyA-oligoU template-primer complex) 2. Briefly, this assay is based on incorporation of [α-32P]UMP into the RNA, precipitation of the products on DE-81 filters (Whatman) and measurement of incorporation rate. The inhibitors were added as described above for NS3 protein activity assays.

NS5BΔ21 (JFH1 isolate of genotype 2a, AB047639) was expressed in E. coli and purified on Ni-NTA-agarose column. Briefly, the sequence encoding NS5BΔ21 was amplified by PCR using the plasmid pJFH1 (a kind gift of Prof. C.M. Rice and Apath L.L.C.) as a template and oligonucleotides 1 (5’-AATGAATTCCATGTCATACTCCTGGAC-3’) and 2 (5’-AATCTCGAGGCGGGGTCGGGCGCGC-3’) as primers. The resulting product (verified by sequencing) was cloned into the bacterial expression vector pET-21d-2c 2. The protein was expressed and purified as above.

The compounds were tested in two assays: primer-dependent (polyA-oligoU complex) and de novo (based on a 3'-UTR of (-)-strand of the virus genome and referred to as (-)IRES). For the de novo activity assay the template was obtained by amplification of the 3'UTR of (-) genome strand using oligonucleotides 3 (5'-TTGTAATACGACTCACTATAGGGTGCACGGTCTACGAGACC-3') and 4 (3'-GACCTGCCCCTAATAGGGCG-3'), where the underlined sequence represents T7 RNA polymerase promoter, in vitro transcription with an 5X MEGAscript T7 Kit (Thermo Fisher Scientific) and purification of the product 3. Enzymatic activity assay conditions were as described

in 4. Briefly, the reaction mixture was subjected to a formaldehyde agarose gel 5, and the radioactivity was visualized on a Packard Cyclone Storage Phosphor System.

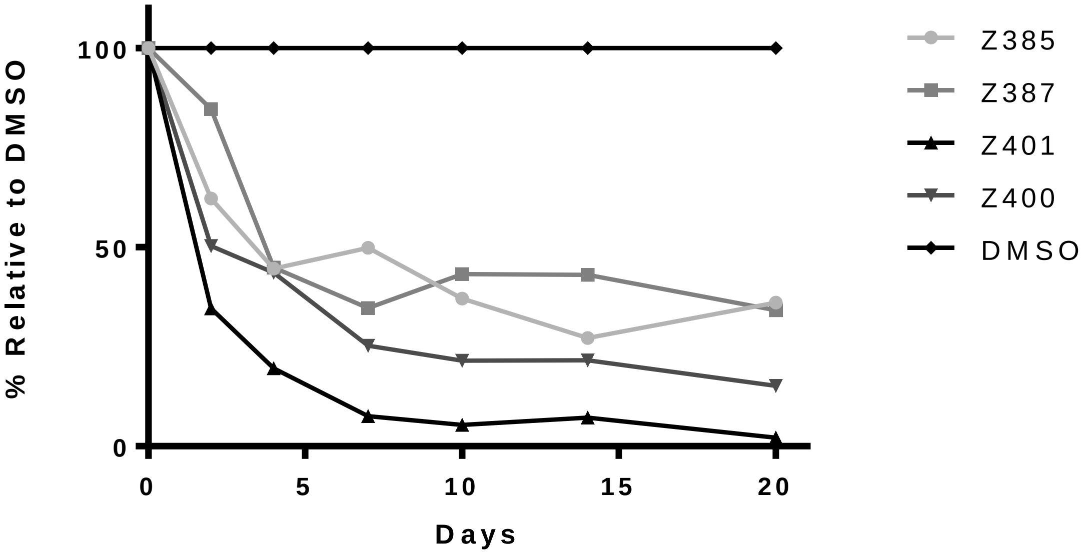

Figure S1: Huh7-J17 cells were cultured in the presence of 3 µM drug as shown for 20 days. Every 3 to 4 days, cells were passaged in the presence of the drugs and the luciferase readings (RLU) determined from 20000 cells and presented as % relative to those from DMSO-treated cells.

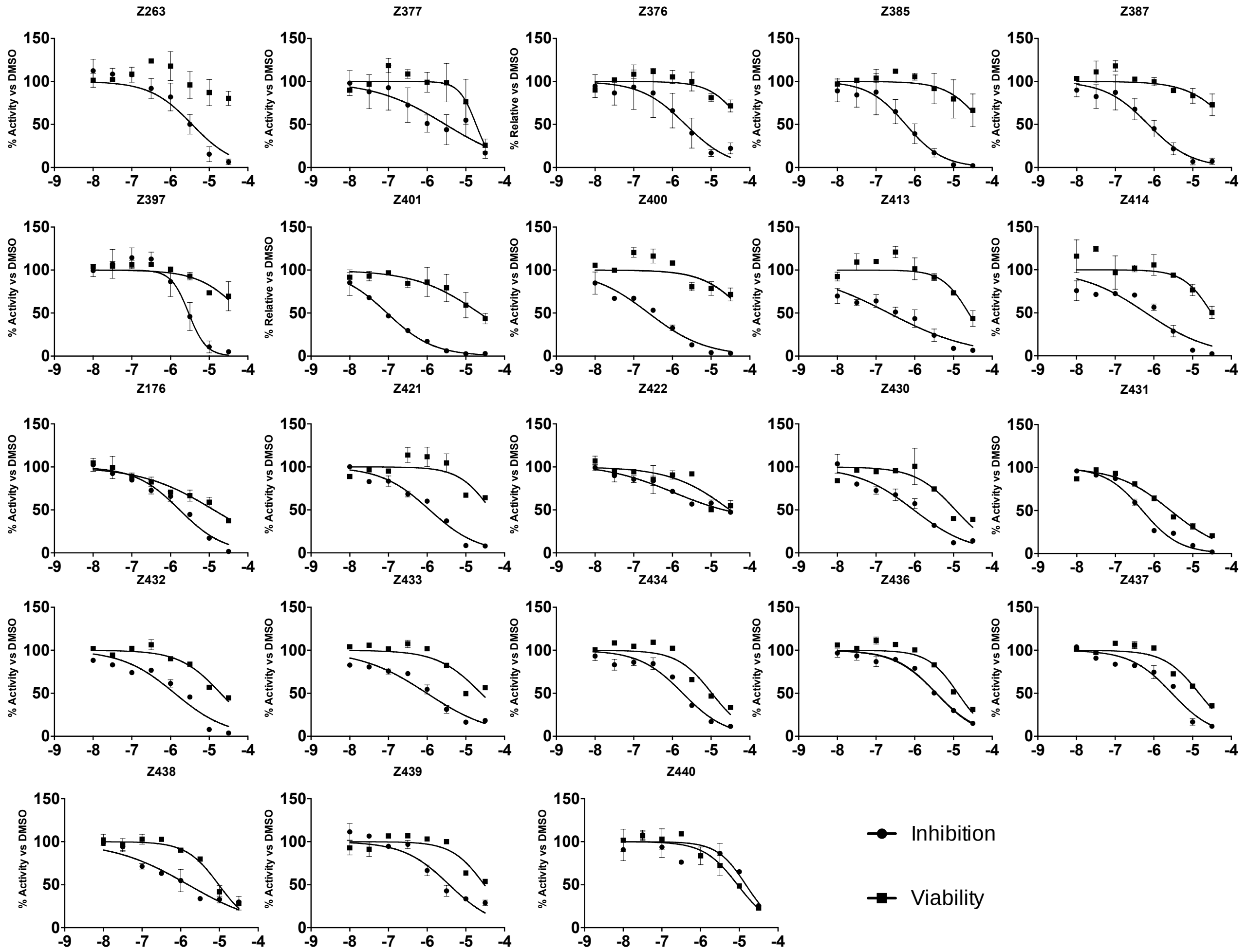

Figure S2: Huh7 cells were pre-treated for 1 h and then infected with JFH1 (gt2a) virus in the presence of different concentration drugs for 3 h. Then, the inoculum was replaced with fresh medium containing compounds. After 3 d viral inhibition was measured evaluating SEAP levels as described. In parallel, cell viability was measured as described in the manuscript. Data are presented as % activity (inhibition or cell viability) relative to the DMSO-treated cells. x-axis – Molar concentration of the compound expressed in a logarithmic scale.

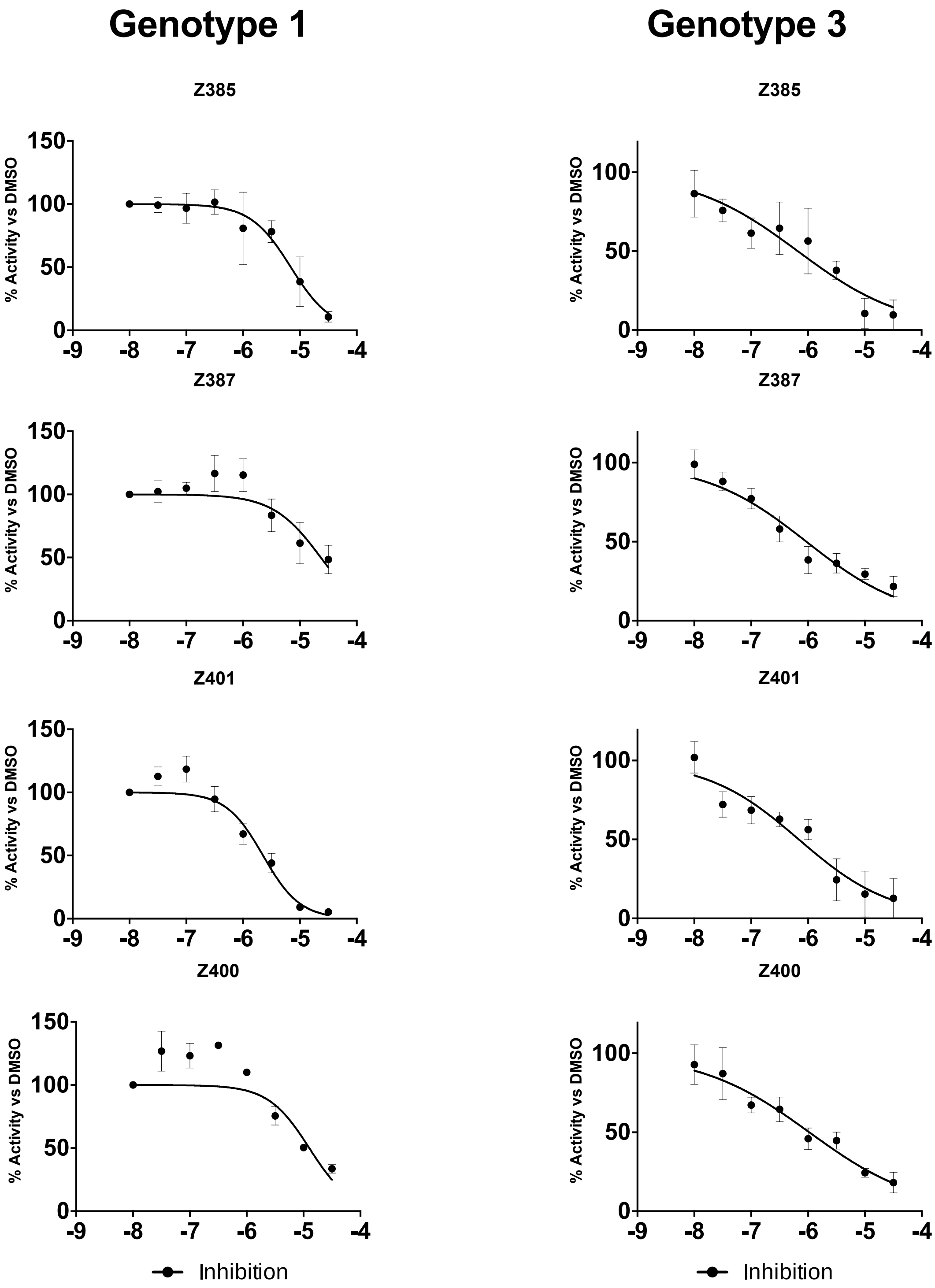

Figure S3: Huh7 cells stably harbouring the subgenomic I389/NS3-3'/LucUbiMeo/ET replicon (gt1b, isolate Con1) were plated and incubated with the compounds at different concentration for 72 h before measurement of the firefly luciferase. Huh7.5-Sec14L2 cells were electroporated with 2 µg of S52-WT-ΔN (gt3a) RNA, plated in the presence of compounds at different concentrations and incubated for 72 h before measuring luciferase. Data are presented as % activity (inhibition or cell viability) relative to the DMSO-treated cells. x-axis – Molar concentration of the compound expressed in a logarithmic scale.

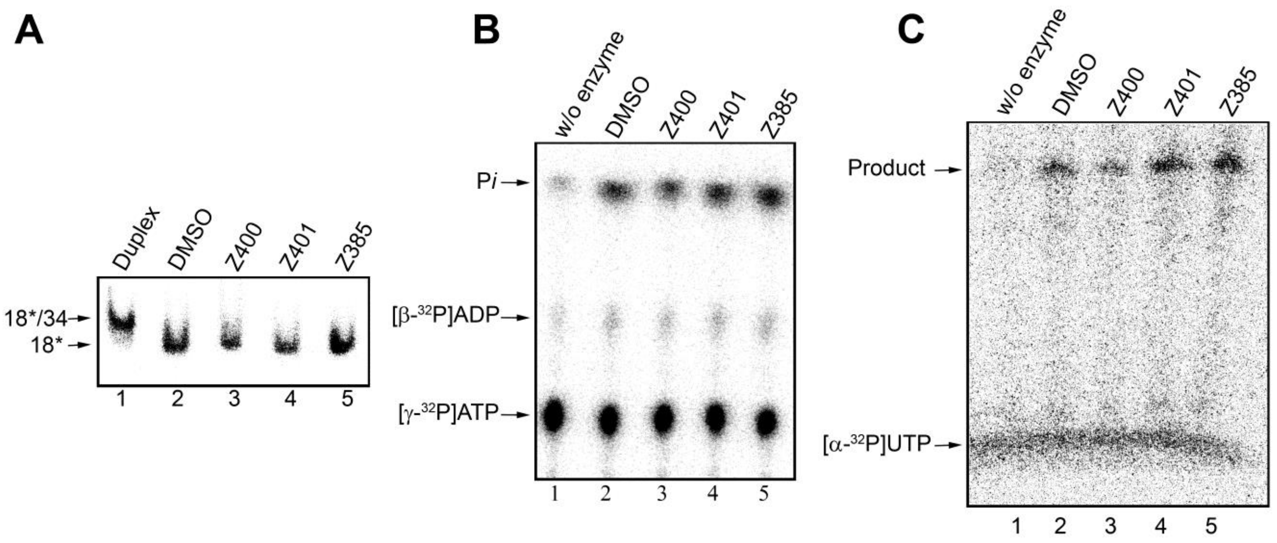

Figure S4: NS3 helicase (A) and NTPase (B) activities as well as NS5B *de novo* RNA-dependent RNA polymerase activity (C) were measured in the absence (DMSO) or presence of the compounds at 50 µM concentration as described above in the "Enzymatic activity" section of the Supplementary materials.

References

1 Tunitskaya, V. L. *et al.* Inhibition of the helicase activity of the HCV NS3 protein by symmetrical dimeric bis-benzimidazoles. *Bioorg Med Chem Lett* **21**, 5331-5335 (2011).

2 Ivanov, A. V. *et al.* Development of the system ensuring a high-level expression of hepatitis C virus nonstructural NS5B and NS5A proteins. *Protein Expr Purif* **48**, 14-23 (2006).

3 Simister, P. *et al.* Structural and functional analysis of hepatitis C virus strain JFH1 polymerase. *J Virol* **83**, 11926-11939 (2009).

4 Ivanov, A. V. *et al.* Hepatitis C virus NS5A protein modulates template selection by the RNA polymerase in in vitro system. *FEBS Lett* **583**, 277-280 (2009).

5 Sambrook, J., Fritsch, E. F. & Maniatis, T. *Molecular cloning: a laboratory manual*. 2nd edn, (Cold Spring Harbor Laboratory Press, 1989).
